# RNA-seq-based evaluation of bicolor tepal pigmentation in Asiatic hybrid lilies (*Lilium* spp.)

**DOI:** 10.1186/s12864-016-2995-5

**Published:** 2016-08-11

**Authors:** Kazuma Suzuki, Tomohiro Suzuki, Takashi Nakatsuka, Hideo Dohra, Masumi Yamagishi, Kohei Matsuyama, Hideyuki Matsuura

**Affiliations:** 1 Faculty of Agriculture, Hokkaido University, N9W9, Kita-ku, Sapporo, 060-8589 Japan; 2 Research Institute of Green Science and Technology, Shizuoka University, 836 Ohya, Suruga-ku, Shizuoka, 422-8529 Japan; 3 Faculty of Agriculture, Shizuoka University, 836 Ohya, Suruga-ku, Shizuoka, 422-8529 Japan; 4 Research Faculty of Agriculture, Hokkaido University, N9W9, Kita-ku, Sapporo, 060-8589 Japan; 5 Present address: Center for Bioscience Research and Education, Utsunomiya University, 350 Mine-machi, Utsunomiya, Tochigi 321-8505 Japan

**Keywords:** Anthocyanin, Flower color pattern, De novo assembly, LhMYB12, R2R3-MYB, Transcriptional regulation, WD40

## Abstract

**Background:**

Color patterns in angiosperm flowers are produced by spatially and temporally restricted deposition of pigments. Identifying the mechanisms responsible for restricted pigment deposition is a topic of broad interest. Some dicots species develop bicolor petals, which are often caused by the post-transcriptional gene silencing (PTGS) of chalcone synthase (*CHS*) genes. An Asiatic hybrid lily (*Lilium* spp.) cultivar Lollypop develops bicolor tepals with pigmented tips and white bases. Here, we analyzed the global transcription of pigmented and non-pigmented tepal parts from Lollypop, to determine the main transcriptomic differences.

**Results:**

De novo assembly of RNA-seq data yielded 49,239 contigs (39,426 unigenes), which included a variety of novel transcripts, such as those involved in flavonoid-glycosylation and sequestration and in regulation of anthocyanin biosynthesis. Additionally, 1258 of the unigenes exhibited significantly differential expression between the tepal parts (false discovery rates <0.05). The pigmented tepal parts accumulated more anthocyanins, and unigenes annotated as anthocyanin biosynthesis genes (e.g., *CHS*, *dihydroflavonol 4-reductase*, and *anthocyanidin synthase*) were expressed 7–30-fold higher than those in non-pigmented parts. These results indicate that the transcriptional regulation of biosynthesis genes is more likely involved in the development of bicolor lily tepals rather than the PTGS of *CHS* genes. In addition, the expression level of a unigene homologous to *LhMYB12*, which often regulates full-tepal anthocyanin pigmentation in lilies, was >2-fold higher in the pigmented parts. Thus, *LhMYB12* should be involved in the transcriptional regulation of the biosynthesis genes in bicolor tepals. Other factors that potentially suppress or enhance the expression of anthocyanin biosynthesis genes, including a *WD40* gene, were identified, and their involvement in bicolor development is discussed.

**Conclusions:**

Our results indicate that the bicolor trait of Lollypop tepals is caused by the transcriptional regulation of anthocyanin biosynthesis genes and that the transcription profile of *LhMYB12* provides a clue for elucidating the mechanisms of the trait. The tepal transcriptome constructed in this study will accelerate investigations of the genetic controls of anthocyanin color patterns, including the bicolor patterns, of *Lilium* spp.

**Electronic supplementary material:**

The online version of this article (doi:10.1186/s12864-016-2995-5) contains supplementary material, which is available to authorized users.

## Background

Lilies are among the most important floricultural crops, owing to their large flowers with unique and diverse colors. The genus *Lilium* consists of >90 species, which are classified into several sections [1, 2], and since species belonging to the same section have relatively high interspecific crossing abilities, interspecific hybridization is the principal method of lily breeding. Among the resulting hybrids, the Asiatic hybrid lilies (*Lilium* spp.) are one of the main groups and are derived from interspecific crosses among the species of section Sinomartagon, which are mainly distributed in East Asia [3].

In lilies, flower color is a commercially important characteristic and much interest has been placed in cultivars that bear flowers with unique colors. Asiatic hybrid lilies, specifically, exhibit large variations in color hue that result from the accumulation of anthocyanins and carotenoids, which result in pink and yellow/orange coloration, respectively, or red coloration with the combination of anthocyanins and orange carotenoids [4–6]. Flavonols, flavones, and cinnamic acid derivatives (CADs) in higher plants are colorless flavonoid or phenylpropanoid compounds having ultraviolet-absorbing characteristics and, in floral organs, these show co-pigmentation effects with anthocyanins [7–9]. In the lily tepals, high amounts of CADs accumulate, whereas flavonols and flavones are often scarcely present [10]. In addition to the wide variation in color hue, the Asiatic hybrid lilies also exhibit variation in color patterns, including the occurrence of several types of spots [11] and bicolor phenotypes, in which two distinct colors occur on individual tepals [4]. For example, the Asiatic hybrid lily cultivar Lollypop has bicolor (pink and white) tepals, in which anthocyanin pigments are heavily accumulated in the upper tepals but less so in the bases.

Anthocyanins are among the most studied and best understood compounds in plant science, and their metabolic pathways have been extensively described (Additional file 1: Figure S1) [12]. The activity of anthocyanin biosynthesis enzymes is primarily controlled at the transcriptional level and is regulated by complexes that consist of the R2R3-MYB and basic helix-loop-helix (bHLH) transcription factors and WD40 proteins (hereafter, MBW complexes) [13, 14]. In angiosperm flowers, large variations in color patterns are observed, owing to the spatially and temporally restricted deposition of anthocyanin pigments, and color pattern variations include bicolor phenotypes, vein-associated anthocyanin pigmentation (venation), stripes and spots, and light-induced pigment accumulation on exposed petal surfaces (bud-blush). Clarifying these mechanisms is a topic of broad interest [15, 16]. The mechanisms of restricted pigment deposition have been characterized in some model plants, and studies have shown that individual species possess multiple *R2R3-MYB* genes that are responsible for regulating the biosynthesis of anthocyanins in flower petals. In petunias, for example, *AN2* generates fully pigmented petals, whereas *PURPLE HAZE* (*PHZ*) determines bud-blush, and *DEEP PURPLE* (*DPL*) causes venation in the flower tubes [17]. In snapdragon, *Rosea1* and *Rosea2* determine whether the petals are fully pink but have different activities, and *Venosa* regulates venation [18, 19]. Furthermore, monkeyflowers (*Mimulus* spp.) have two *R2R3-MYB* genes, *PELAN* and *NEGAN*, which control anthocyanin production in the petal lobes and in the spots of the nectar guide, respectively [20]. In non-model lilies, several *R2R3-MYBs* have been shown to control anthocyanin pigmentation, together with *LhbHLH2*, in flowers [21–24]. For example, *LhMYB12* controls anthocyanin pigmentation in whole tepals [22], *Latvia* allele of *LhMYB12* determines splatter-type tepal spots [23], and *LrMYB15* regulates a bud-blush phenotype in *Lilium regale* [24]. These observations in both model plants and lilies indicate that R2R3-MYB transcription factors are principally involved in color patterning.

Mechanisms other than the regulation by R2R3-MYB transcription factors cause bicolor patterns. The bicolor phenotypes of star and marginal picotee (white margin, pigmented center) petunias are caused by the post-transcriptional gene silencing (PTGS) of the *chalcone synthase* (*CHS*) *A* gene, which is one of the anthocyanin biosynthesis genes, in white areas [25, 26]. Another type of marginal picotee petunias that exhibit a pigmented margin and white center is not caused by the down-regulation of anthocyanin biosynthesis genes. At the white centers, increased accumulations of flavonols and *flavonol synthase* (*FLS*) transcripts have been detected [27], which indicates that there is competition between the enzymes FLS and dihydroflavonol-4-reductase (DFR) for the common substrate, dihydroflavonol, and that flavonol biosynthesis is more dominant than anthocyanin biosynthesis at the white centers. These findings indicate that several mechanisms are involved in bicolor generation. However, the mechanisms of bicolor tepal development in lilies have yet to be analyzed.

*Lilium* spp. have a huge genome (33–36 Gb) [28], often exhibit gametophytic self-incompatibility, and have long life cycles (3–7 years from sowing to anthesis). Although a few molecular linkage maps have been developed, using PCR-based markers [29, 30], high-density genetic maps are lacking. Mutant and tagging lines are not developed yet, and both stable and transient transformation are still challenging. Thus, the genetic analysis of agricultural traits in lilies is difficult. To date, several biosynthesis and regulatory genes that are involved in anthocyanin biosynthesis in lily flowers have been identified [21, 22, 31–34]; however, the overall molecular mechanisms that underlie tepal pigmentation remains limited; e.g., no sequence information of WD40 proteins or anthocyanin-glycosylating enzymes. In addition, although several negatively regulating transcription factors have been reported in other species [35–37], such information is not available in lilies.

Enrichment of genetic resources, including transcriptome sets, is essential in order to provide effective tools for further extensive and intensive research on more complicated flower traits in lilies. The whole transcriptome sequencing based on next-generation sequencing (NGS) and de novo assembly enable transcriptome studies of non-model organisms without reference sequences [38, 39]. The strategy has been widely applied to non-model plants, e.g., to floricultural crops, in order to obtain mass sequence data for the molecular mechanisms responsible for color diversity [40–42]. Because of the huge genome size, whole genome sequencing is currently unavailable in lilies. Thus, de novo transcriptome sequencing has been applied to *Lilium* spp., and several NGS studies have already been published. More than two thousand simple sequence repeats (SSR) were detected in expressed sequences used to develop SSR markers [43, 44], the global transcription of lily bulb meristems after cold-treatment was profiled to understand the molecular regulation of vernalization [45, 46], and genome-wide transcription was analyzed to clarify the genetic response to cold-stress [47]. However, there have been no NGS studies concerning color diversification in lily tepals.

In the present study, whole transcriptome sequencing was conducted using the bicolor Asiatic hybrid lily cultivar Lollypop. RNA samples from pink (pigmented) and white (non-pigmented) tepal parts were sequenced using Illumina NGS, and the gene expression levels of the two tepal regions were compared. The objectives of the present study were to sequence the whole transcriptome of lily tepals and to define the main transcriptomic differences between the two tepal parts, in order to characterize the mechanisms involved in complicated bicolor traits.

## Results and discussion

### Qualitative and quantitative analyses of anthocyanins and CADs

**Fig. 1:**
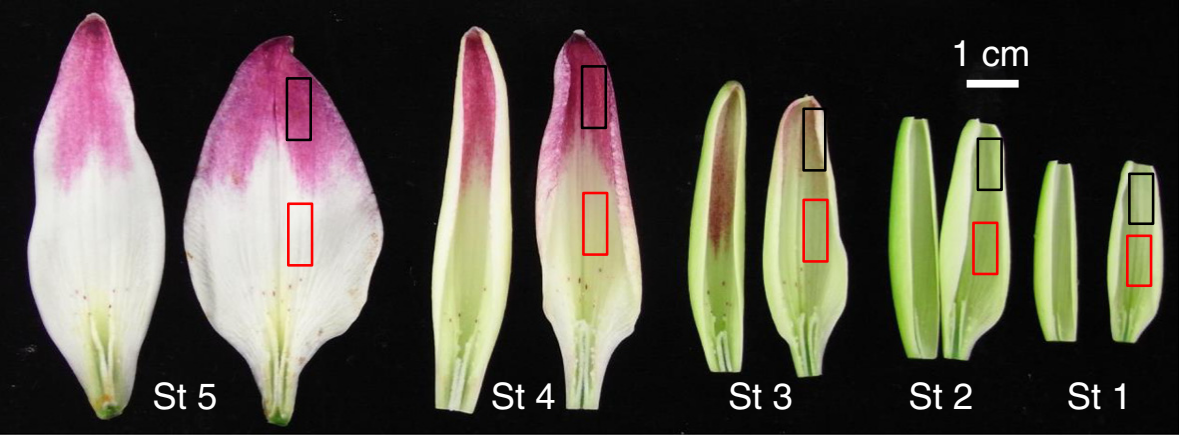
Tepal development of the Asiatic hybrid lily Lollypop. Inner tepal (right) and outer tepal (left) at each stage are shown. Flower bud development stages (St) are as follows: St 1, no anthocyanin pigmentation; St 2, red spots appeared on tepal bases; St 3, beginning of pigmentation in upper tepals; St 4 (1 d before anthesis), maximum pigmentation; St 5, blossoming. Scale bar = 1 cm. Colored boxes indicate tepal parts used for the experiments (black boxes–upper tepals, red boxes–tepal bases)

The Lollypop cultivar exhibited bicolor tepals, with upper tepals that were pink and tepal bases that were white or pale yellow with red raised spots, and the color of the tepals during floral development were as follows: stage (St) 1, tepals were not pigmented yet; St 2, red spots appeared on bases; St 3, pigmentation began in upper tepals; St 4 (one day before anthesis), the content of tepal anthocyanin was highest; and St 5, flowering (Fig. 1). First, we measured the amount of pigments accumulated in the pink upper tepals and the white bases using high-performance liquid chromatography (HPLC). The segments of upper tepals and tepal bases were cut out from the same inner tepals at St 4, and pigments were evaluated with three replicates (each replicate derived from a different flower). The Lollypop tepals included a single anthocyanin pigment, and its retention time and absorbance spectrum were identical to those of cyanidin 3-*O*-β-rutinoside (Additional file 1: Figure S2). However, flavones and flavonols were not detected (data not shown). Furthermore, at least two major peaks were detected by absorbance at 340 nm (Additional file 1: Figure S2), and a wavelength scan of each of the peaks suggested that they included a caffeic acid moiety. Because such products are derived from cinnamic acid, we hereafter refer to these compounds as CADs [10, 27]. These products were further separated by silica gel column chromatography and thin-layer chromatography (TLC), and one of the major compounds was identified using ^1^H- and ^13^C-NMR as 1-*O*-β-D-caffeoylglucose [48].

**Fig. 2:**
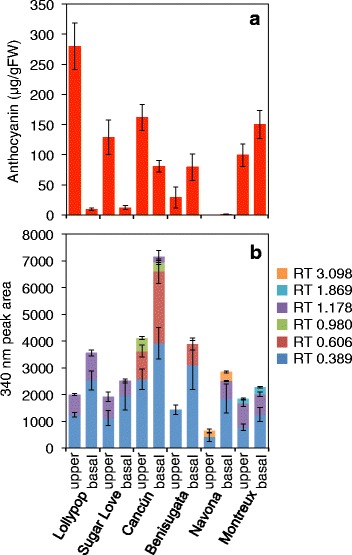
Anthocyanins and cinnamic acid derivatives in the tepals of six Asiatic hybrid lily (*Lilium*) cultivars. **a** Anthocyanin content. **b** The six cinnamic acid derivatives were distinguished by HPLC retention time (RT, min). Values and error bars indicate the means ± standard error (n = 3). FW: fresh weight

When the amounts of anthocyanins and CADs of the pink upper tepals and white bases were compared, high amounts of anthocyanins were detected in the upper tepals, whereas much less was found in the bases (Fig. 2a). In contrast, the amount of CADs was higher in the bases than in the upper tepals (Fig. 2b), confirming that the accumulation profiles of the two tepal parts were different.

In the marginal picotee cultivars of petunia, which exhibit pigmented margins and white centers, a dramatic increase in flavonol accumulation is detected in the white centers [27]. To verify whether the higher accumulation of CADs in the bases specifically occurred in bicolor lily cultivars, the anthocyanin and CAD contents of five additional cultivars, which included two other bicolor cultivars, were examined (Fig. 2). In the bicolor cultivars Sugar Love (pink upper tepals and white bases) and Cancún (red upper tepals and yellow bases), more anthocyanins were found in upper tepals, in accordance with their color. In the Cancún cultivar, because of pink abaxial surface of inner tepals, a relatively small difference was observed in the anthocyanin content of the upper tepals and tepal bases. Meanwhile, in the non-bicolor cultivars Benisugata (red tepals) and Montreux (pink tepals), more anthocyanins were found in the bases, and no or very little anthocyanins were detected in the white tepal cultivar Navona.

Among the six Asiatic hybrid cultivars, six types of CADs were detected and were distinguished by retention time (RT). The total amounts of CADs were higher in the tepal bases than in upper tepals in all of the tested cultivars. The most abundant compound (RT = 0.389 min) was commonly detected among the six cultivars, and its amounts were also higher in tepal bases (Fig. 2b). These results indicate that the higher accumulation of CADs in the bases of lily tepals is not specific to bicolor cultivars.

### Segregation of the bicolor trait in F_1_ progeny

To determine the genetic background of the bicolor trait, segregation of the bicolor trait was analyzed using F_1_ plants that were derived from crosses between Lollypop and another Asiatic hybrid lily cultivar (Blackout) that has tepals fully pigmented by anthocyanins. Wild species and cultivars of *Lilium* are heterozygous, and thus, traits often segregate in F_1_ progenies. Of the 240 F_1_ plants, 226 exhibited bicolor tepals, and 14 exhibited fully pigmented tepals. This segregation was skewed to the Lollypop parent (bicolor trait) and did not fit the segregation ratios of 1:1 or 3:1, which indicates that the bicolor trait is not inherited in a Mendelian fashion and is controlled by at least several genes.

### Sequencing, de novo assembly, and detection of differentially expressed genes (DEGs)

To elucidate the genetic controls of complicated traits, we performed RNA-seq for the pink upper part and white basal part of St 3 tepals with two replicates. Two replicates were derived from distinct plants and, in each replicate, segments of upper tepals and tepal bases were collected from three inner tepals of a single flower. These samples were analyzed in a single run on the Illumina MiSeq to obtain relatively highly expressed sequences. Approximately 50 million raw reads (~24 million fragments) were obtained as a result and de novo assembly yielded 39,426 unigenes (Additional file 1: Table S1).

We compared the expression of the 39,426 unigene in the upper tepals and tepal bases and 1258 exhibited significantly differential expression (false discovery rate [FDR] <0.05, Additional file 2: Table S2). The positive and negative log2 Fold Change (LogFC) values respectively indicated the up- and down-regulation of the unigenes in upper tepals, relative to the bases, and indicated that 665 unigenes (53 %) were up-regulated in upper tepals.

### Unigenes related to anthocyanin and CAD accumulation

One of the aims of the present study was to obtain the sequences of all the genes involved in anthocyanin and CAD biosyntheses in *Lilium*. These biosynthesis pathways are branched from the general phenylpropanoid pathway, and a number of enzymes are involved (Additional file 1: Figure S1). *Phenylalanine ammonia-lyase* (*PAL*), *CHS*, *chalcone isomerase* (*CHI*), *flavanone 3-hydroxylase* (*F3H*), *flavonoid 3′-hydroxylase* (*F3′H*), *DFR*, and *anthocyanidin synthase* (*ANS*) genes have been identified in *Lilium* but the other genes have not. Thus, the unigene sequences of putatively encoded proteins were BLASTed against the Swiss-Prot database and 15, 31, and three unigenes in Lollypop sequence database were predicted to encode phenylpropanoid, anthocyanin, and cinnamic acid biosynthesis enzymes, respectively. In addition, 27 candidates were predicted to encode proteins that are required for flavonoid sequestration in vacuoles, including members of the *multidrug and toxic compound extrusion* (*MATE*) gene family and *glutathione S-transferase* (*GST*) genes (Table 1) [49, 50]. All of the main genes necessary for anthocyanin and CAD biosyntheses were included in the Lollypop transcriptome. Among the candidates for *CHI*, *F3H*, *F3′H*, *DFR*, and *ANS*, unigenes with relatively high FPKM (Fragments Per Kb per Million mapped reads) values (hereafter, major unigenes) had high sequence similarity to the corresponding genes isolated from the other Asiatic hybrid lily cultivar or *L. speciosum* [10, 31, 34]. In some cases, unigenes did not correspond well to previously isolated genes in lilies: Unigene c29955_g1 (annotated as *PAL*) consisted of three *PAL* gene sequences (*LhPAL1*, *LhPAL2*, and *LhPAL3*) and unigene c30110_g1 (annotated as *CHS*) contained *LhCHSa* and *LhCHSb* sequences (Additional file 1: Figure S3). It was expected that these gene sequences were not distinguished during de novo assembly.

**Table 1:** Annotation and expression of unigenes involved in anthocyanin and cinnamic acid derivative biosyntheses and transport

Putative gene (according to Swiss-Prot)	Trinity transcript id	Homologous gene in *Lilium*	Blastp top hit	Length	Identity	E-value	FPKM (expression) in		logFC	logCPM	FDR
							Upper tepals	Tepal bases			
Phenylpropanoid pathway genes											
PAL	c29955_g1	*LhPAL1/2/3*	phenylalanine ammonia lyase [Lilium hybrid division I]	729	99 %	0	631.93	472.92	0.41	10.09	0.7580164
PAL	c36922_g1	*LhPAL3*	phenylalanine ammonia lyase [Lilium hybrid division I]	114	95 %	2E-64	44.13	5.04	3.14	3.09	2.07E-05
PAL	c30134_g17		phenylalanine ammonia lyase, partial [Musa acuminata AAA Group]	436	81 %	0	12.30	19.98	−0.65	4.32	0.6531477
PAL	c30134_g11		phenylalanine ammonia-lyase [Betula pendula]	115	64 %	6E-44	10.26	14.29	−0.37	1.37	0.9748477
PAL	c30134_g1		phenylalanine ammonia-lyase, partial [Rhizophora apiculata]	191	84 %	4E-113	8.62	14.20	−0.62	2.34	0.7807557
C4H	c26579_g1		cinnamate-4-hydroxylase [Canarium album]	507	88 %	0	1030.58	803.78	0.37	10.53	0.6668013
C4H	c25090_g1		cinnamate 4-hydroxylase, partial [Tulipa fosteriana]	330	90 %	0	3.54	10.24	−1.43	2.61	0.1219183
4CL	c29891_g1		4-hydroxycinnamoyl-CoA ligase 2 [Coffea arabica]	518	70 %	0	344.61	485.55	−0.39	9.02	0.6668013
4CL	c27769_g1		4-coumarate-CoA ligase-like protein [Arabidopsis thaliana]	561	66 %	0	112.74	114.24	−0.01	7.48	1
4CL	c27493_g1		coumaroyl-CoA ligase [Vitis vinifera]	522	72 %	0	91.97	63.02	0.57	6.96	0.6023584
4CL	c26975_g1		4-coumarate--CoA ligase-like 7 [Vitis vinifera]	572	62 %	0	34.27	32.43	0.09	5.87	0.9889067
4CL	c26242_g1		4-coumarate--CoA ligase-like 9 [Vitis vinifera]	482	61 %	0	14.85	26.40	−0.79	4.82	0.4774231
4CL	c29687_g1		4-coumarate--CoA ligase-like 6 [Glycine soja]	582	62 %	0	12.42	13.44	−0.07	4.41	1
4CL	c20611_g1		4cl [Lilium hybrid cultivar]	549	97 %	0	5.39	16.11	−1.53	4.22	0.04040995
4CL	c25512_g2		4-coumaroyl-coenzyme A ligase [Allium cepa]	225	65 %	3E-99	6.45	6.72	0.03	2.02	1
Cinnamic acid derivative biosynthesis genes											
C3H	c28376_g1		4-coumarate 3-hydroxylase [Eucalyptus globulus]	508	77 %	0	6.19	7.07	−0.17	2.89	0.9748477
HCT	c27695_g1		Hydroxycinnamoyl-CoA:quinate hydroxycinnamoyltransferase [Zostera marina]	431	49 %	2E-125	34.28	37.59	−0.12	5.65	0.9748477
HCT	c30288_g1		hydroxycinnamoyl transferase [Nicotiana benthamiana]	324	63 %	6E-147	5.02	46.05	−3.14	4.45	0.0001381333
Anthocyanin biosynthesis genes											
CHS	c30110_g1	*LhCHSa/b*	chalcone synthase [Lilium hybrid division I]	267	99 %	0	1209.02	421.80	1.47	9.77	0.01588364
CHS	c29657_g1	*LhCHSc*	chalcone synthase C [Lilium hybrid division I]	249	98 %	1E-173	267.20	63.11	2.10	7.05	0.001083655
CHS	c29657_g2	*LhCHSc*	chalcone synthase C [Lilium hybrid division I]	249	97 %	1E-174	48.68	104.39	−1.12	6.01	0.1175434
CHS	c30110_g2		chalcone synthase, partial [Tulipa fosteriana]	242	72 %	4E-120	52.81	72.32	−0.47	5.73	0.6531477
CHS	c29920_g1		chalcone synthase, partial [Tulipa fosteriana]	389	78 %	0	34.11	19.43	0.65	4.42	0.4774231
CHI	c28538_g1	*LsCHIb*	chalcone isomerase, partial [Lilium speciosum]	210	99 %	4E-135	1232.45	816.96	0.60	9.77	0.4774231
CHI	c28136_g1	*LsCHIa*	chalcone isomerase [Lilium speciosum]	234	97 %	2E-156	276.09	178.28	0.63	7.91	0.4774231
CHI	c27139_g1		Chalcone-flavanone isomerase family protein isoform 1 [Theobroma cacao]	420	50 %	2E-133	34.06	22.96	0.56	5.63	0.5573998
CHI	c23754_g1		Chalcone-flavanone isomerase family protein isoform 3, partial [Theobroma cacao]	299	64 %	6E-85	12.87	16.08	−0.32	3.55	0.7828791
F3H	c28413_g1	*LhF3H*	flavanone 3-hydroxylase [Lilium hybrid division I]	370	97 %	0	666.13	330.01	1.03	9.21	0.1196772
F3'H	c27194_g1	*LhF3'H*	flavonoid 3'-hydroxylase [Lilium hybrid division I]	515	99 %	0	195.25	33.85	2.54	7.58	1.38E-06
DFR	c30307_g2	*LhDFR*	dihydroflavonol 4-reductase [Lilium hybrid division I]	376	98 %	0	3143.14	1550.13	0.98	11.27	0.1175434
DFR	c24884_g1		dihydroflavonol-4-reductase-like [Elaeis guineensis]	348	68 %	3E-163	87.02	71.25	0.28	6.49	0.7807557
DFR	c29645_g2		cinnamoyl-CoA reductase 1-like [Phoenix dactylifera]	303	70 %	2E-138	35.55	34.81	−0.02	4.50	1
DFR	c27848_g1		dihydroflavonol-4-reductase [Vitis vinifera]	334	73 %	0	15.05	15.08	0.01	4.00	1
DFR	c12034_g1		Dihydroflavonol-4-reductase [Aegilops tauschii]	92	57 %	3E-27	11.78	4.96	1.20	0.42	0.6318245
DFR	c27774_g2		Dihydroflavonol-4-reductase [Aegilops tauschii]	104	57 %	2E-34	8.23	5.01	0.70	1.25	0.6668013
DFR	c9045_g1		Bifunctional dihydroflavonol 4-reductase/flavanone 4-reductase [Morus notabilis]	86	65 %	9E-32	0.00	10.40	−5.20	−0.17	0.1055062
ANS	c26135_g1	*LhANS*	anthocyanidin synthase [Lilium hybrid division I]	362	98 %	0	1767.61	876.34	1.02	10.75	0.1055062
ANS	c28679_g2		Gibberellin 3-beta-dioxygenase 4 [Morus notabilis]	316	42 %	2E-76	62.99	243.34	−1.97	7.33	0.0006236071
ANS	c27368_g1		Leucoanthocyanidin dioxygenase [Morus notabilis]	377	62 %	1E-160	68.14	50.96	0.42	6.02	0.6531477
ANS	c19298_g1		Leucoanthocyanidin dioxygenase [Gossypium arboreum]	362	67 %	7E-175	8.51	29.34	−1.79	4.38	0.002704819
3GT	c35034_g1		UDP-glucose: anthocyanidin 3-O-glucosyltransferase [Tulipa fosteriana]	484	78 %	0	1650.78	1337.40	0.31	11.18	0.7580164
3GT	c16413_g1		anthocyanidin 3-O-glucosyltransferase [Tulipa fosteriana]	450	71 %	0	120.55	90.36	0.41	7.09	0.6531477
3GT	c29247_g1		anthocyanidin 3-O-glucosyltransferase [Zea mays]	489	50 %	8E-137	19.03	20.65	−0.10	4.92	0.9889067
3GT	c26782_g1		UDP glucose:flavonoid 3-O-glucosyltransferase [Pyrus x bretschneideri]	109	54 %	3E-32	21.19	16.46	0.45	2.17	0.9367663
3GT	c26782_g5		anthocyanidin 3-O-glucosyltransferase [Tulipa fosteriana]	346	57 %	6E-115	11.65	13.85	−0.18	3.53	0.9889067
3RT	c25725_g1		anthocyanidin-3-glucoside rhamnosyltransferase [Petunia x hybrida]	465	55 %	0	2144.38	1507.37	0.51	11.53	0.6023584
7GT	c29610_g1		scopoletin glucosyltransferase-like [Phoenix dactylifera]	489	60 %	0	53.84	80.60	−0.64	5.55	0.4774231
7GT	c28503_g1		UDP-glycosyltransferase 1 [Linum usitatissimum]	418	44 %	6E-107	16.58	25.74	−0.57	4.50	0.6318245
7GT	c26782_g3		phenylpropanoid:glucosyltransferase 2, partial [Nicotiana tabacum]	377	49 %	1E-117	9.78	10.58	−0.09	3.36	0.9889067
Transporters of anthocyanins and/or cinnamic acid derivatives, to vacuole											
MATE	c27999_g1		MATE efflux family protein [Arabidopsis thaliana]	535	62 %	0	483.91	412.47	0.25	9.61	0.8141854
MATE	c29991_g1		MATE efflux family protein [Arabidopsis thaliana]	507	72 %	0	106.41	103.68	0.03	7.40	1
MATE	c30035_g1		transparent testa 12 protein [Zea mays]	489	69 %	0	50.45	116.90	−1.18	7.11	0.06782638
MATE	c25329_g1		transparent testa 12 protein [Zea mays]	527	78 %	0	66.84	94.90	−0.50	7.10	0.6318245
MATE	c23411_g1		MATE efflux family protein LAL5-like isoform X1 [Phoenix dactylifera]	517	73 %	0	37.53	41.30	−0.16	5.82	0.9243257
MATE	c1112_g1		MATE efflux family protein 5 [Phoenix dactylifera]	510	69 %	0	14.64	21.87	−0.52	4.84	0.6668013
MATE	c19647_g1		MATE efflux family protein 5 [Vitis vinifera]	481	55 %	0	13.65	13.72	−0.05	3.77	1
MATE	c23623_g1		transparent testa 12 protein [Zea mays]	480	78 %	0	9.71	8.01	0.32	3.81	0.8141854
MATE	c27478_g1		MATE efflux family protein [Theobroma cacao]	258	65 %	1E-108	6.83	10.52	−0.50	3.06	0.6668013
MATE	c12223_g2		MATE efflux family protein 5-like [Musa acuminata subsp. malaccensis]	212	78 %	2E-112	3.74	8.87	−1.26	1.79	0.3152999
MATE	c17372_g1		MATE efflux family protein 5-like [Phoenix dactylifera]	148	74 %	3E-63	7.98	3.35	1.24	0.91	0.4774231
MATE	c29524_g1		MATE efflux family protein 5-like [Elaeis guineensis]	196	77 %	3E-103	5.02	5.95	−0.40	0.93	0.9243257
GST	c24611_g1		glutathione S-transferase [Vitis amurensis]	228	57 %	9E-88	1251.26	959.39	0.40	9.90	0.7580164
GST	c27339_g1		glutathione S-transferase [Allium cepa]	215	56 %	7E-82	554.35	774.28	−0.49	8.90	0.6318245
GST	c28555_g1		elongation factor 1, partial [Lilium regale]	418	89 %	0	162.71	190.06	−0.22	8.00	0.8141854
GST	c27591_g1		glutathione S-transferase T1-like [Musa acuminata subsp. malaccensis]	244	73 %	1E-122	170.25	170.91	0.00	7.01	1
GST	c25698_g2		glutathione S-transferase [Tulipa fosteriana]	229	84 %	2E-133	87.22	128.32	−0.83	5.88	0.3152999
GST	c24296_g1		elongation factor 1-gamma 2 [Zea mays]	417	78 %	0	90.46	123.33	−0.44	7.27	0.6318245
GST	c25698_g1		glutathione S-transferase [Arachis hypogaea]	207	71 %	8E-94	90.79	120.45	−0.40	5.57	0.6668013
GST	c22668_g1		glutathione S-transferase 2 [Oryza sativa Japonica Group]	221	69 %	8E-103	93.72	80.32	0.20	6.25	0.9131831
GST	c27057_g1		glutathione s-transferase [Elaeis guineensis]	221	67 %	4E-104	20.61	21.78	−0.09	4.04	0.9889067
GST	c27057_g2		glutathione s-transferase [Elaeis guineensis]	258	65 %	2E-98	28.04	5.32	2.46	3.63	0.100767
GST	c21745_g1		Glutathione S-transferase Phi class [Zostera marina]	221	65 %	1E-96	3.32	19.01	−2.49	2.80	0.003065782
GST	c20342_g1		glutathione S-transferase family protein [Populus trichocarpa]	149	64 %	1E-53	8.57	12.71	−0.54	1.72	0.7580164
GST	c27063_g1		Tetrachloro-P-hydroquinone reductive dehalogenase [Zea mays]	266	71 %	3E-146	9.96	10.03	0.10	3.65	0.9889067
GST	c19621_g1		glutathione S-transferase [Musa acuminata AAA Group]	111	66 %	6E-43	13.16	2.27	2.40	1.02	0.2599001
GST	c33779_g1		Glutathione S-transferase Phi class [Zostera marina]	99	60 %	1E-35	0.00	10.44	−5.65	0.05	0.1057929

Most wild species and cultivars of *Lilium*, including Lollypop, mainly accumulate cyanidin 3-*O*-β-rutinoside, although some also accumulate cyanidin 3-*O*-β-rutinoside-7-*O*-β-glucoside [5]. In the present study, several unigenes were annotated as *anthocyanidin 3-O-glucosyltransferase* (*3GT*), *anthocyanidin-3-glucoside rhamnosyltransferase* (*3RT*), and *anthocyanidin-3-rutinoside 7-glucosyltransferase* (*7GT*), among which unigenes c35034_g1 (*3GT*) and c25725_g1 (*3RT*) exhibited relatively high FPKM values.

Among the unigenes involved in phenylpropanoid and anthocyanin biosyntheses, the major unigenes annotated as *LhPAL3* (c36922_g1), *LhCHSa/b* (c30110_g1), *LhCHSc* (c29657_g1), and *LhF3′H* (c27194_g1) were highly expressed in the upper tepals (FDR <0.05). These expression profiles were coincident with higher accumulation of anthocyanin pigments in upper tepals. One *ANS*-annotated unigene (c28679_g2), which showed relatively high FPKM values, was significantly high in its expression in tepal bases (FDR <0.05). However, according to NCBI BLAST (http://blast.ncbi.nlm.nih.gov/Blast.cgi), c28679_g2 showed no hits for ANS and was annotated as gibberellin 3-ß-dioxygenase. Thus, it is still unknown whether the protein encoded by this unigene has anthocyanidin synthase activity.

### Unigenes related to the regulation of anthocyanin accumulation

In higher plants, the expression of anthocyanin biosynthesis genes is commonly regulated by MBW complexes [13, 14]. Although the functions of LhMYB12 and LhbHLH2 in regulating anthocyanin biosynthesis in the tepals of *Lilium* spp. are well evaluated, WD40 orthologues have not been isolated. To detect *WD40* orthologues in *Lilium*, we BLASTed the amino acid sequence of AtTTG1 (WD40 in *Arabidopsis*) against the Lollypop sequence database. Then, unigene c2514_g1 was obtained, which exhibited 78 % amino acid identity to PhAN11 (WD40 in *Petunia*, data not shown).

*R2R3-MYB* genes constitute the largest MYB gene family in plants, with 125 *R2R3-MYB* genes in *Arabidopsis* and 109 in rice, and regulate plant-specific processes, including the biosynthesis of secondary metabolites [51, 52]. In order to conduct a comprehensive search for *R2R3-MYB* genes in *Lilium*, we BLASTed the R2R3 repeat motif sequence [51] against the Lollypop tepal transcriptome. As a result, 51 MYB unigenes were detected, including two MYB3R, 44 R2R3-MYB, three R3-MYB, and two unusual MYB genes that had two or more repeats (Table 2). We have isolated 14 *R2R3-MYB* cDNA sequences (*LhMYB1*–*12*, *LhMYB15*, and *LhMYB16*) from wild species and cultivars of *Lilium*. Of the 14 *R2R3-MYB* sequences, 10 were highly similar to 11 Lollypop unigenes (Table 2), although four (*LhMYB4*, *6*, *9*, and *11*) were not represented in the Lollypop sequence database.

**Table 2:** Annotation and expression of unigenes showing the homology with R2R3-MYB, R3-MYB, and MYB3R genes

Type and sub-group (SG)^a)^	Trinity transcript id	Homologous gene in Lilium	Homologous gene in Arabidopsis	Homologous gene in other species	Blastp top hit	Length	Identity	E-value	FPKM (expression) in		logFC	logCPM	FDR
									Upper tepals	Tepal bases			
MYB3R type													
	c19823_g1		*AtMYB3R-1*		myb-related protein 3R-1-like isoform *X*2 [Phoenix dactylifera]	242	74 %	2E-115	4.24	7.13	−0.61	1.90	1
	c27498_g5		*AtMYB3R-3*,*3R-5*		myb-related protein 3R-1-like [Musa acuminata subsp. malaccensis]	502	50 %	7E-154	10.74	11.67	−0.11	4.65	1
R2R3-MYB type													
SG 1	c10428_g1		*AtMYB30,94,96*	*Am306*	R2R3-MYB transcriptional factor, partial [Lilium hybrid division I]	124	75 %	2E-42	2.96	2.18	0.30	−0.14	1
SG 1	c19652_g1	*LhMYB7*	*AtMYB30*,*94*,*96*	*Am306*	R2R3-MYB transcriptional factor, partial [Lilium hybrid division I]	193	99 %	2E-112	6.94	3.00	1.20	1.56	0.5056056
SG 1	c19652_g2	*LhMYB7*	*AtMYB30*,*94*,*96*	*Am306*	R2R3-MYB transcriptional factor, partial [Lilium hybrid division I]	109	100 %	1E-63	1.72	5.00	−1.19	0.26	0.9933287
SG 1	c23337_g2	*LhMYB5*	*AtMYB30*,*94*,*96*	*Am306*	R2R3-MYB transcriptional factor, partial [Lilium hybrid division I]	309	98 %	6E-141	7.72	14.20	−0.85	3.12	0.5775627
SG 2	c2281_g1		*AtMYB13*,*14*,*15*		putative MYB transcription factor [Rosa rugosa]	89	83 %	3E-48	10.39	7.12	0.65	1.35	1
SG 2	c8990_g1		*AtMYB13*,*14*,*15*		myb-like protein Myb15 [Citrus maxima]	83	83 %	8E-47	1.73	8.46	−2.10	−0.42	0.9933287
SG 2	c12438_g1		*AtMYB13*,*14*,*15*		myb-related protein Zm1-like [Elaeis guineensis]	259	51 %	1E-64	14.05	12.72	3.19	2.36	0.004292067
SG 2	c18397_g1		*AtMYB13*,*14*,*15*		Myb-related protein Zm1 [Aegilops tauschii]	206	51 %	1E-43	4.60	4.21	0.21	1.28	1
SG 2	c18397_g2		*AtMYB13*,*14*,*15*		myb-related protein Myb4-like [Brachypodium distachyon]	43	72 %	2E-14	5.15	1.96	1.99	0.35	0.4220982
SG 2	c35333_g1		*AtMYB13*,*14*,*15*		MYB transcription factor [Zea mays]	121	87 %	3E-62	1.58	4.09	−1.24	−0.07	1
SG 2	c36958_g1		*AtMYB13*,*14*,*15*		myb-related protein Myb4 [Zea mays]	98	69 %	7E-35	1.81	1.79	−0.05	−0.66	1
SG 4	c10735_g1	*LhMYB3*	*AtMYB3*,*4*,*7*,*32*	*FaMYB1*, *Am308*, *Am330*	R2R3-MYB transcriptional factor [Lilium hybrid division I]	119	97 %	5E-81	5.83	3.37	0.91	1.16	1
SG 4	c25442_g1	*LhMYB8*	*AtMYB3*,*4*,*7*,*32*	*FaMYB1*, *Am308*, *Am330*	R2R3-MYB transcriptional factor [Lilium hybrid division I]	214	99 %	2E-156	273.93	218.16	0.31	7.76	1
SG 4	c25442_g2		*AtMYB3*,*4*,*7*,*32*	*FaMYB1*, *Am308*, *Am330*	myb-related protein 308-like [Elaeis guineensis]	252	69 %	1E-119	72.64	61.13	0.25	6.28	1
SG 6	c22900_g1	*LhMYB12*	*PAP1*,*PAP2*	*PhAN2*	transcription factor R2R3-MYB [Lilium hybrid division I]	162	95 %	6E-97	276.37	216.83	0.30	7.89	1
SG 6	c24386_g1	*LrMYB15*	*PAP1*,*PAP2*	*PhAN2*	transcription factor R2R3-MYB [Lilium regale]	242	88 %	4E-155	7.23	15.11	−1.05	3.20	0.3424106
SG 6	c24386_g2	*LhMYB16*	*PAP1*,*PAP2*	*PhAN2*	R2R3-MYB transcriptional factor, partial [Lilium hybrid division I]	240	84 %	5E-75	5.47	8.71	−0.69	2.32	0.9933287
SG 7	c25442_g3		*AtMYB11*,*12*,*111*	*ZmP*	MYB12 [Arabidopsis thaliana]	287	70 %	1E-72	30.92	11.36	1.49	3.84	0.3424106
SG 9	c10215_g1		*AtMYB17*	*AmMIXTA*	Myb domain protein 17 isoform 2 [Theobroma cacao]	72	66 %	1E-19	0.00	4.63	−3.28	−0.92	1
SG 9	c10215_g2		*AtMYB17*	*AmMIXTA*	myb domain protein 17 [Zostera marina]	80	91 %	2E-46	n.d.	n.d.	n.d.	n.d.	n.d.
SG 9	c22634_g1	*LhMYB10*	*AtMYB17*	*AmMIXTA*	R2R3-MYB transcriptional factor, partial [Lilium hybrid division I]	245	98 %	1E-143	7.57	11.78	−0.62	2.96	0.9933287
SG 9	c22634_g2		*AtMYB17*	*AmMIXTA*	Transcription factor [Morus notabilis]	107	99 %	5E-72	13.11	16.98	−0.29	2.07	1
SG 13	c4482_g2		*AtMYB55*,*86*		putative transcription factor MYB55 [Arabidopsis thaliana]	105	96 %	3E-62	2.67	1.34	0.69	−0.51	1
SG 13	c16280_g2		*AtMYB55*,*86*		MYB transcription factor MYB89 [Glycine max]	107	48 %	6E-25	2.58	4.98	−0.64	0.04	1
SG 13	c35026_g1		*AtMYB55*,*86*		transcription factor MYB34-like [Nicotiana tomentosiformis]	86	90 %	2E-50	2.17	1.79	0.42	−0.55	1
SG 18	c19459_g1		*AtMYB65*,*101*	*HvGAMYB*	transcription factor GAMYB [Vitis vinifera]	480	43 %	7E-119	5.55	4.82	0.16	2.80	1
SG 18	c32967_g1		*AtMYB65*,*101*	*HvGAMYB*	transcription factor GAMYB-like [Musa acuminata subsp. malaccensis]	115	67 %	2E-49	2.44	2.07	0.41	0.41	1
SG 19	c7757_g1	*LhMYB2*	*AtMYB21*,*24*	*Am305*, *Am340*, *PhEOBII*	R2R3-MYB transcriptional factor [Lilium hybrid division I]	191	100 %	4E-142	2609.05	2336.57	0.16	11.12	1
SG 19	c23501_g1	*LhMYB1*	*AtMYB21*,*24*	*Am305*, *Am340*, *PhEOBII*	LhMyb [Lilium hybrid division I]	192	99 %	5E-131	2929.08	2745.59	0.09	11.22	1
SG 21	c7270_g1		*AtMYB105*,*117*	*MlRCP1*	myb-like protein Q [Musa acuminata subsp. malaccensis]	156	52 %	3E-18	3.36	0.32	3.04	0.02	0.3424106
SG 21	c16635_g1		*AtMYB105*,*117*	*MlRCP1*	MYB143 protein [Gossypium hirsutum]	73	70 %	0.0000004	20.94	0.95	4.16	2.15	0.00079
SG 22	c12801_g2		*AtMYB44*, *70*, *73*, *77*		transcription factor MYB44-like [Vitis vinifera]	410	51 %	2E-100	23.84	29.80	−0.29	5.21	1
SG 22	c13168_g1		*AtMYB44*, *70*, *73*,* 77*		MYB transcription factor MYB178 [Glycine max]	75	78 %	3E-24	0.00	3.69	−3.20	−0.93	1
SG 22	c21029_g1		*AtMYB44*, *70*, *73*, *77*		sucrose responsive element-binding protein [Elaeis guineensis]	205	61 %	7E-72	5.17	13.50	−1.35	2.23	0.3424106
SG 22	c21511_g2		*AtMYB44*, *70*, *73*, *77*		Myb domain protein 73 [Theobroma cacao]	222	59 %	4E-55	62.90	87.74	−0.45	5.24	0.9953253
SG 22	c37985_g1		*AtMYB44*, *70*, *73*,* 77*		transcription factor MYB44-like [Musa acuminata subsp. malaccensis]	92	53 %	5E-24	0.73	1.16	−0.13	−0.93	1
SG 23	c1544_g1		*AtMYB1*		myb-related protein B-like [Gossypium raimondii]	135	65 %	3E-50	2.32	3.46	−0.30	0.18	1
SG 23	c26297_g1		*AtMYB1*		transcription factor MYB86 [Cucumis sativus]	331	57 %	5E-116	14.64	13.84	0.06	3.86	1
SG 25	c14426_g1		*AtMYB98*		transcription factor MYB98-like [Prunus mume]	77	61 %	1E-23	9.25	1.90	2.24	−0.35	0.7101734
	c14549_g1		*AtMYB20*, *85*	*PhODO1*	protein ODORANT1 [Sesamum indicum]	125	54 %	4E-36	4.46	4.21	−0.01	0.35	1
	c14549_g2		*AtMYB20*, *85*	*PhODO1*	myb-related protein 315-like [Nicotiana tomentosiformis]	67	88 %	4E-40	0.00	7.78	−4.67	−0.44	0.3424106
	c16655_g1		*AtMYB20*, *85*	*PhODO1*	protein ODORANT1-like [Elaeis guineensis]	263	64 %	6E-118	3.83	5.67	−0.47	1.75	1
	c28204_g2		*AtMYB88*,*124*		Myb124, putative isoform 1 [Theobroma cacao]	509	45 %	3E-119	6.92	9.76	−0.19	3.60	1
	c24798_g1		*AtMYB91*/*AS1*	*AmMYBPHAN*	transcription factor AS1 [Populus euphratica]	345	69 %	3E-165	10.58	7.27	0.52	3.44	0.9933287
R3-MYB type													
	c24227_g1		*AtMYBL2*		transcription repressor MYB4-like [Nelumbo nucifera]	157	42 %	3E-37	4.82	3.86	0.41	0.79	1
	c24227_g2		*AtMYBL2*		myb-related protein 308-like [Phoenix dactylifera]	157	43 %	3E-34	28.20	3.00	3.29	2.42	0.00079
	c18278_g2		*AtCAPRICE*	*PhMYBx*	transcription factor CPC-like [Nelumbo nucifera]	90	62 %	6E-23	5.87	4.64	0.28	−0.13	1
'Unusual' MYB genes with two or more repeats													
	c28856_g1		*AtCDC5*		cell division cycle 5-like protein [Arabidopsis thaliana]	1049	73 %	0	12.74	13.64	−0.06	5.40	1
	c23757_g4		*AtMYB4R1*		myb-like protein L [Phoenix dactylifera]	197	74 %	3E-98	6.93	5.83	−0.20	1.21	1

^a)^The grouping follows [51]

R2R3-MYBs are further classified into subgroups, and the genes belonging to the same subgroup often share similar functions [51, 53]. Of the 44 R2R3-MYBs expressed in Lollypop tepals, 39 were clustered into 13 subgroups. The Lollypop unigenes c22900_g1 (LhMYB12), c24386_g1 (LrMYB15), and c24386_g2 (LhMYB16) were assigned to subgroup 6, the members of which are often involved in the regulation of anthocyanin biosynthesis. LhMYB12 usually regulates full-tepal pigmentation in lilies [22]. In contrast, LrMYB15 has been shown to regulate the anthocyanin pigmentation responsible for bud-blush in *L. regale* [24], although its function in Asiatic hybrid lilies is unknown, and the functions of LhMYB16 have yet to be investigated.

In addition, the three unigenes c10735_g1 (LhMYB3), c25442_g1 (LhMYB8), and c25442_g2 were designated as subgroup 4 R2R3-MYBs, which are suppressors and often inhibit anthocyanin or lignin biosyntheses [54, 55]; however, the functions of LhMYB3 and LhMYB8 are not yet understood. R3-MYBs are short MYBs that only include the R3 repeat, and AtMYBL2 and AtCAPRICE in *Arabidopsis* and PhMYBx in petunias are R3-MYBs that suppress anthocyanin biosynthesis [17, 35, 36]. In the present study, the three unigenes c24227_g1, c24227_g2, and c18278_g2 were identified as R3-MYBs (Table 2).

In lilies, the two bHLH genes LhbHLH1 (petunia JAF13 type) and LhbHLH2 (petunia AN1 type) have been shown to regulate anthocyanin biosynthesis, especially LhbHLH2 [21]. We BLASTed these sequences against the Lollypop transcriptome. As a result, four short unigenes (c16322_g1–4) exhibited similarities of >90 % to the amino acid sequence of LhbHLH1 in Montreux. Therefore, these unigenes should be considered a single gene, even though they were not assembled during de novo assembly. Similarly, the two unigenes c7085_g1 and c27198_g1 exhibited high similarities to the N- and C-terminal halves of the LhbHLH2 sequence in Montreux, respectively. Thus, the LhbHLH2 cDNA sequence should be split into two unigenes. These results indicate that the orthologous genes of both LhbHLH1 and LhbHLH2 are expressed in Lollypop tepals, although the FPKM values were higher in c7085_g1 and c27198_g1 (LhbHLH2) than in c16322_g1–4 (LhbHLH1; data not shown).

### Transcription in upper tepals and tepal bases during floral development

**Fig. 3:**
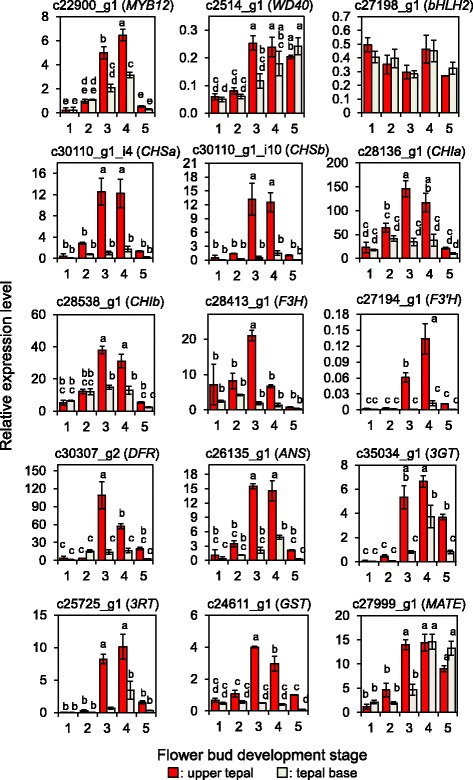
Relative expression of 15 unigenes in Asiatic hybrid lily Lollypop tepals during floral development. The 15 target genes included c22900_g1 (*LhMYB12*), c2514_g1 (*WD40*), c27198_g1 (*LhbHLH2*), c30110_g1_i4 (*LhCHSa*), c30110_g1_i10 (*LhCHSb*), c28136_g1 (*CHIa*), c28538_g1 (*CHIb*), c28413_g1 (*LhF3H*), c27194_g1 (*LhF3′H*), c30307_g2 (*LhDFR*), c26135_g1 (*LhANS*), c35034_g1 (*3GT*), c25725_g1 (*3RT*), c24611_g1 (*GST*), and c27999_g1 (*MATE*), and their expression was normalized using *ACTIN* expression. Floral development stages (1–5) are shown in Fig. 1. Values and error bars indicate the means ± standard error (n = 3). The same letters above the columns indicate that the values are not statistically significant (*p* <0.05) by Tukey’s HSD

To determine whether the expression of MBW complex genes corresponded to the development of tepal color, we performed quantitative reverse transcription-PCR (qRT-PCR) using the total RNA of the upper tepals and tepal bases collected at various stages of floral development (St 1–5). The upper and basal tepal segments were collected from a single inner tepal and each stage included three replicates, each of which was derived from a different flower. The expression of c22900_g1 (*LhMYB12*) increased with the progression of floral development showing the highest at St 4, and was >2-fold higher in the upper tepals than in the tepal bases at St 3 and 4 (Fig. 3). This expression pattern was coincident with the accumulation of anthocyanins, which also increased at St 3 and 4 and was higher in the upper tepals. The expression of c2514_g1 (*WD40*) increased gently during tepal development, although it was also higher in upper tepals than in tepal bases at St 3. In contrast, c27198_g1 (*LhbHLH2*) was consistently expressed, with no significant differences in expression between development states or tepal location.

In addition, the expression of major unigenes involved in anthocyanin biosynthesis was also evaluated by qRT-PCR. We found that the expression of unigenes annotated as *LhCHSa*, *LhCHSb*, *LhF3H*, *LhF3′H*, *LhDFR*, and *LhANS* were higher at St 3 and/or 4 during tepal development and were 7–30-fold higher in the upper tepals than in tepal bases at St 3 when anthocyanin pigments started to accumulate in upper tepals (Fig. 3). The expression of c28136_g1 (*CHIa*), c28538_g1 (*CHIb*), c35034_g1 (*3GT*), c25725_g1 (*3RT*), and c24611_g1 (*GST*) was higher at St 3 and/or 4 during floral development and was 2–12-fold higher in the tepal bases than in upper tepals (Fig. 3). c27999_g1 (*MATE*) exhibited high expression at St 3, 4, and 5 but no clear difference of the expression between the two tepal parts at St 4 and 5. These results indicate that most of the unigenes involved in anthocyanin biosynthesis and sequestration, other than *MATE*, show higher expression in the pigmented upper part at St 3 and St 4.

St 3 tepals were used for RNA-seq in this study. All of DEGs (e.g., c30110_g1 and c27194_g1, Table 1) showed clear difference in expression at St 3 in qRT-PCR analysis but not all of unigenes showing significant difference at St 3 by qRT-PCR were detected as DEGs. This may be due to the low number of fragments (~24 million fragments) in our RNA-seq analysis, which should lower the power to detect DEGs.

### Factors involved in bicolor development

Some of the mechanisms involved in the development of bicolor petals have been identified in model plants. For example, the star and marginal picotee (white margin, pigmented center) patterns in petunias are caused by PTGS of *CHS-A* in the white petal regions, rather than changes in the expression of anthocyanin biosynthesis genes [25, 26]. In the present study, unigenes annotated as *LhCHSa*, *LhCHSb*, *LhF3H*, *LhF3′H*, *LhDFR* and *LhANS* exhibited >7-fold higher expression in upper tepals than in the tepal bases (Fig. 3), which indicated a strong correlation between the expression of anthocyanin biosynthesis genes and anthocyanin pigmentation, and the coordinated down-regulation of these genes in the tepal bases indicates that the bicolor trait is not caused by PTGS.

Another mechanism of bicolor development has been reported in the marginal picotee pattern of petunias with pigmented margins and white centers; at the white center, the increase of flavonol production should prevent anthocyanin production [27]. Although no flavonols and flavones were detected in lily tepals, during the present study, high amounts of CADs accumulated and reached greater levels in the tepal bases than in the upper tepals (Fig. 2). This observation suggests that competition for *p*-coumaroyl CoA, which occurs at a branching point of the anthocyanin and CAD biosynthesis pathways and is a common substrate of CHS and shikimate *O*-hydroxycinnamoyltransferase (HCT, Additional file 1: Figure S1), occurs between the biosynthesis of anthocyanins and CADs in lily tepals. This idea is supported by previous studies suggesting that the suppression of *HCT* expression results in an increase in flavonoid biosynthesis and an accumulation of anthocyanins [56]. However, in Lollypop, although a unigene annotated as *HCT* (c30288_g1) exhibited a higher FPKM value in tepal bases at St 3 (Table 1), its transcription at St 3 and 4 were relatively low during floral development (Additional file 1: Figure S4). In addition, higher amounts of CADs in tepal bases were not a specific feature to bicolor cultivars and were also observed in the full-pink and white tepal cultivars (Fig. 2). Furthermore, in petunias, elevated *FLS* expression failed to affect the expression of either *DFR* or *ANS* [27], whereas in lilies, the expression of major unigenes annotated as anthocyanin biosynthesis were all suppressed in non-pigmented regions. Therefore, the active biosynthesis of CADs is not likely responsible for the bicolor trait, and novel mechanisms are likely involved.

Because the expressions of the *LhCHSa*, *LhCHSb*, *LhF3H*, *LhF3′H*, *LhDFR*, and *LhANS* annotated unigenes were coordinately up-regulated in the upper tepals of the Lollypop cultivar, the transcriptional regulation of these biosynthesis genes is most likely to influence the bicolor phenotype. In higher plants, MBW complexes predominantly regulate the transcription of anthocyanin biosynthesis genes, and R2R3-MYB transcription factors have a key role in the spatial and temporal restriction of anthocyanin biosynthesis [13, 14]. Anthocyanin pigmentation is regulated by subgroup 6 R2R3-MYBs in many plant species and by subgroup 5 R2R3-MYBs in Gramineae and Orchidaceae [57, 58]. In the Lollypop tepal transcriptome, three unigenes were assigned to subgroup 6, although none was assigned to subgroup 5. Among the three unigenes in subgroup 6, c22900_g1 (*LhMYB12*) exhibited the highest FPKM (Table 2) and its expression was correlated with pigment deposition, with higher expression at St 3 and 4 of floral development (Fig. 3). Meanwhile, unigenes c24386_g1 (*LhMYB15*) and c24386_g2 (*LhMYB16*) exhibited relatively low FPKM values (Table 2). Thus, c22900_g1 (*LhMYB12*) is more likely to regulate the biosynthesis of anthocyanins in Lollypop tepals. Since LhMYB12 regulates both early (e.g., *CHS*) and late (e.g., *DFR* and *ANS*) biosynthesis genes in Asiatic hybrid lilies [10], c22900_g1 (*LhMYB12*) transcripts might be responsible for the coordinated expression of *LhCHSa*, *LhCHSb*, *LhF3H*, *LhF3′H*, *LhDFR*, and *LhANS* unigenes. In addition, the expression of c22900_g1 (*LhMYB12*) was >2-fold higher in upper tepals than in tepal bases (Fig. 3). In another bicolor cultivar Sugar Love, the expression of *LhMYB12* was >4-fold higher in upper tepals (Additional file 1: Figure S5), whereas a previous study has demonstrated that *LhMYB12* expression was higher in the tepal bases of full-pink tepal cultivar Montreux [22]. Therefore, the expression profiles of c22900_g1 (*LhMYB12*) are correlated with bicolor creation.

### Factors that suppress the transcription of anthocyanin biosynthesis genes

Although the expression of c22900_g1 (*LhMYB12*) was >2-fold different in the upper tepals and tepal bases, the expression of the anthocyanin biosynthesis unigenes exhibited a >7-fold difference (Fig. 3). This pattern suggests that the expression of c22900_g1 is partly responsible for the low expression of biosynthesis genes in tepal bases, although it does not explain it completely, and that other factors contribute to the complete suppression of biosynthesis genes. WD40 proteins stabilize the MBW complexes and increase the activity of the complexes as a result [59–61]. In Lollypop tepals, the expression of c2514 (WD40) was higher in upper tepals at St 3 (Fig. 3). The similar expression profiles of WD40 were also observed in the other bicolor cultivar Sugar Love but were not detected in the full-pink cultivar Montreux (Additional file 1: Figure S5). These observations suggest that the lower expression of c2514 (WD40) in non-pigmented tepal regions is specific to bicolor cultivars and synergistically reduces the expression of the biosynthesis genes in tepal bases.

Putative unigenes that could suppress the expression of anthocyanin biosynthesis genes were identified in the Lollypop transcriptome, e.g., subgroup 4 R2R3-MYBs, R3-MYBs, and *SQUAMOSA PROMOTER BINDING PROTEIN-LIKE* (*SPL*) genes [62] (Table 3). If suppressor genes are involved in bicolor development, their expressions should be higher in tepal bases at St 3 and/or 4. However, qRT-PCR analysis during the floral development indicated that c18278_g2 (R3-MYB) expression was higher in tepal bases at St 5 and the expressions of other unigenes were higher in upper tepals or similar between the two tepal parts (Table 3, Additional file 1: Figure S4). Thus, the functions of these unigenes remain to be determined.

**Table 3:** Expression profiles of unigenes that potentially suppress the expression of anthocyanin biosynthesis genes

Unigene	Homologous gene in lilies	Homologous genes in other species	Putative function	Reference	Expression profile^a^
c10735_g1	*LhMYB3*	*AtMYB4*, *FaMYB1*, *Am308*, *Am330*	Subgroup 4 R2R3-MYB that inhibits anthocyanin or lignin biosyntheses	[54, 55]	Higher in upper tepals at St 3 and 4
c25442_g1	*LhMYB8*	*AtMYB4*, *FaMYB1*, *Am308*, *Am330*	Subgroup 4 R2R3-MYB that inhibits anthocyanin or lignin biosyntheses	[54, 55]	Similar between the two tepal parts
c25442_g2		*AtMYB4*, *FaMYB1*, *Am308*, *Am330*	Subgroup 4 R2R3-MYB that inhibits anthocyanin or lignin biosyntheses	[54, 55]	Similar between the two tepal parts
c24227_g1		*AtMYBL2*	R3-MYB that suppresses anthocyanin biosynthesis	[35]	Higher in upper tepals at St 4
c24227_g2		*AtMYBL2*	R3-MYB that suppresses anthocyanin biosynthesis	[35]	Higher in upper tepals at St 3 and 4
c18278_g2		*AtCAPRICE*, *PhMYBx*	R3-MYB that suppresses anthocyanin biosynthesis	[17, 36]	Higher in tepal bases at St 5
c36339_g1		*AtSPL9*, *AtSPL15*	SPL9 and 15 directly prevent the expression of anthocyanin biosynthetic genes by destabilizing MBW complexes	[62]	Similar between the two tepal parts

^a^Expression during floral development is shown in Additional file 1: Figure S4

### Factors that influence the expression of MYB12

In the Lollypop cultivar, the expression of c22900_g1 (*LhMYB12*) was higher in the upper tepals, whereas a previous study has demonstrated that the expression of *LhMYB12* is higher in the bases of fully pink tepals [22]. In *LhMYB12*, six allelic sequences, Montreux [22], Renoir [63], Landini [63], Vermeer [33], Latvia [23], and Sugar Love [unpublished result] alleles, have been detected in Asiatic hybrid lilies. The sequence of c22900_g1 was identical to that of the Landini allele of *LhMYB12*, which suggests that the Landini allele shows differential expression profiles in the Lollypop and the fully pink cultivar Landini. In addition, Cancún also possesses the Landini allele, while Sugar Love possesses the Sugar Love allele, which demonstrate that not all bicolor cultivars possess the same *LhMYB12* allele. These observations indicate that other factors that function upstream of *LhMYB12* are responsible for the unique expression profiles of *LhMYB12* observed in bicolor cultivars.

The upstream transcription factors of R2R3-MYB are well characterized in the fruits, seeds, and vegetative organs of other species [64–68] but not in flowers. However, Reduced Carotenoid Pigmentation 1 (RCP1) has recently been shown to down-regulate the expression of an anthocyanin biosynthesis activator in *Mimulus* flowers [69]. RCP1 is an R2R3-MYB that belongs to subgroup 21, to which the two Lollypop unigenes c7270_g1 and c16635_g1 were also assigned (Table 2). Since c16635_g1 showed higher expression at St 4 when anthocyanin contents were highest (Additional file 1: Figure S4), their role should be further examined.

In addition to transcriptional regulation by transcription factors, post-transcriptional regulation by microRNA should also be considered. Transcripts of R2R3-MYB that regulate anthocyanin biosynthesis are often targeted by microRNA828 (miR828) [70]. Sequences of miR828 target sites are well conserved in the *R2R3-MYB* genes of dicots and monocots [71], and in the present study, one such sequence was found in c22900_g1 (*LhMYB12*, Additional file 1: Figure S6). Thus, primary-miR828 (pri-miR828) in *Vitis* (VvPri-miR828a) was used in a BLAST search of the Lollypop transcriptome, and a unigene c13793_g1, encoding putative pri-miR828, was obtained. Although the sequences and lengths of pri-miR828 sequences are highly divergent, the 22-bp sequence of the putative miR828 was completely conserved in pri-miR828 sequences (Additional file 1: Figure S6). Differences in the FPKM values of c13793_g1 were small in upper tepals and tepal bases. However, because multiple regulation systems affect microRNA processing efficiency [72], further analyses of c13793_g1 will be necessary.

## Conclusions

In the present study, we analyzed the global transcription in the bicolor lily cultivar Lollypop to determine the main transcriptomic differences in pigmented and non-pigmented tepal parts. We identified and annotated a large number of unigenes, including those for both anthocyanin modification and sequestration, thus providing an excellent platform for future genetic and functional genomic research. In addition, the expression profiles of flower color-related genes in both upper tepals and tepal bases provide new insight into the molecular mechanisms underlying the development of bicolor tepals in Asiatic hybrid lilies. Our results also indicate that the development of bicolor tepals is the result of the transcriptional regulation of anthocyanin biosynthesis genes, rather than PTGS or substrate competition, and moreover, the unique transcription profile of the transcription factor *LhMYB12* indicates that it plays a key role. Therefore, the up- and down-stream factors associated with LhMYB12 function should be further investigated. Fortunately, the tepal transcriptome generated by the present study will accelerate such investigations.

## Methods

### Plant material

The Asiatic hybrid lily (*Lilium* sp.) Lollypop was grown in a greenhouse (unheated and natural photoperiod) at the experimental farm of Hokkaido University, Sapporo, Japan. Flower tepals for transcriptome sequencing were collected at St 3 (Fig. 1, the flower stages followed [31]). To analyze the segregation of the bicolor trait, Lollypop was crossed with the Asiatic hybrid lily Blackout, and their F_1_ progeny were grown under the same conditions.

### Pigment measurement

Anthocyanins and related compounds were extracted with a solvent mixture of methanol, acetic acid, and water (4:1:5, v:v:v), and the extracts were filtered through a ZORBAX Eclipse Plus C18 column (Agilent Technologies, Tokyo, Japan). The extract solution of each sample was then analyzed using HPLC with an Agilent 1290 infinity system (Agilent Technologies). A linear gradient of 10-60 % of solvent B (1.5 % H_3_PO_4_, 20 % acetic acid, and 25 % acetonitrile) in solvent A (1.5 % H_3_PO_4_) was run over a 9-min period. The anthocyanins and CADs were identified on the basis of absorption spectra obtained using a photodiode array detector, and pigment quantity was determined on the basis of absorption values at wavelengths of 340 nm (CADs) and 530 nm (anthocyanins).

### Structural determination of CADs

Tepals of Lollypop (15 g) were soaked in 100 % ethanol, and after filtering the ethanol solution with filter paper (No. 1; Toyo, Tokyo, Japan), the filtrate was concentrated in vacuo. The resulting material was dissolved in 1 L 80 % ethanol to obtain the precipitate, and after filtering the solution with filter paper, the supernatant was again concentrated in vacuo and was subsequently purified using silica gel column chromatography (methanol/chloroform, 1:15, v/v) and preparative TLC (methanol/chloroform, 1:15, v/v) to obtain caffeoylglucose. The silica gel column chromatography was performed using 50 g of Kanto silica gel 60 N (60–210 mesh; Kanto Kagaku, Tokyo, Japan) and the preparative TLC was performed using Merck silica gel 60 F_254_ (Art 5554; Merck, Darmstadt, Germany). Nuclear magnetic resonance (NMR) spectra were recorded using a Bruker AM-500 (^1^H at 500 MHz; Bruker, Billerica, MA, USA) and a JEOL JNM-EX 270 FT-NMR system (^1^H at 270 MHz; ^13^C at 67.5 MHz; JEOL, Tokyo, Japan).

### Transcriptome sequencing

Four cDNA samples were prepared from total RNA isolated from the pink upper tepals and white basal tepals of Lollypop at St 3. The upper tepal and tepal base samples included two replicates, which were collected from two different plants. Total RNA was extracted from the tepal parts using TriPure Isolation Reagent (Roche, Basel, Switzerland) and Fruit-mate for RNA Purification (TaKaRa, Shiga, Japan), according to the manufacturer’s protocols. To prevent contamination by genomic DNA, the RNA was treated with deoxyribonuclease I (Thermo Fisher Scientific, Yokohama, Japan) and purified using an RNeasy Mini Kit (Qiagen, Tokyo, Japan). The RNA integrity was assessed using an Agilent 2100 Bioanalyzer (Agilent Technologies), and the samples were prepared for transcriptome analyses using a SureSelect Strand Specific RNA Library Prep Kit (Agilent Technologies), following the manufacturer’s instructions. First, poly(A) RNA was purified from 4 μg total RNA using oligo (dT) magnetic beads, and then the poly(A) RNA was chemically fragmented at 94 °C for 4 min. Next, first- and second-strand cDNA was synthesized using fragmented RNA as templates, and the resulting double-stranded cDNA was end repaired, adenylated, and ligated using SureSelect Oligo Adaptor (Agilent Technologies). These products were selectively amplified using PCR and were sequenced (75 bp, paired-end) using a MiSeq System (Illumina Inc., San Diego, California, USA). Then, we obtained approximately 24 million fragments (paired-end reads), which consisted of 7,589,796 (upper tepals, replicate 1), 4,653,736 (upper tepals, replicate 2), 5,002,031 (tepal bases, replicate 1), and 6,909,815 (tepal bases, replicate 2) fragments.

### Raw data processing and de novo assembly

Raw data processing, base calling, and quality control were performed using RTA, OLB, and CASAVA (Illumina), according to the manufacturer’s pipeline. The output sequence quality was inspected using FastQC (http://www.bioinformatics.babraham.ac.uk/projects/fastqc/), and the reads were cleaned using cutadapt (version 1.8.1) [73] to trim low-quality ends (<QV30), the 76th nucleotides, and adapter sequences, and to discard reads shorter than 50 bp. Then, 48,897,598 high quality reads (98 % of the raw data) were selected. The clean reads were then assembled de novo using the Trinity program version r20140413p1 [74], yielding 49,239 contigs that clustered into 39,426 subcomponents (i.e., unigenes). The unigene sizes were 201 to 10,772 bp, with a mean length of 427 bp, N50 length of 1228 bp, and total combined length of 29,260,585 bp (Additional file 1: Table S1).

### Differential expression analysis

We identified DEGs using scripts bundled with the Trinity package according to “Trinity Transcript Quantification” (https://github.com/trinityrnaseq/trinityrnaseq/wiki/Trinity-Transcript-Quantification) and “Differential Expression Analysis Using a Trinity Assembly” (https://github.com/trinityrnaseq/trinityrnaseq/wiki/Trinity-Differential-Expression) with a default setting to process the strand-specific RNA-seq data generated from the upper tepals and tepal bases, with two biological replicates from each tepal part. The RNA-seq reads were aligned to full-transcript sequences using bowtie 1.0.0 [75]. Transcript abundance was then estimated by RSEM 1.2.11 [76], and DEGs were identified using edgeR 3.6.8 [77], with trimmed mean of M-values (TMM) normalization. The FDR values were recalculated from the edgeR *p*-values by considering only the unigenes listed in Tables 1, 2, and Additional file 2: Table S2.

### Functional annotation and identification of orthologous genes

For functional annotation, the sequences of putatively encoded proteins were BLASTed against the public protein databases (Swiss-Prot [78], Pfam [79], eggNOG [80], and Gene Ontology [81]) using the BLASTX algorithm and a typical cut off value of E <0.00001. Of the 39,426 unigenes, 24,835 (63 %) were annotated, and the number of unigenes annotated by the Swiss-Prot, Pfam, eggNOG, and GO databases were 21,649; 24,835; 8575; and 16,084, respectively. The Swiss-Prot and Pfam databases accounted for a large proportion of the annotations, and the unigenes that were annotated with gene descriptions from eggNOG and GO databases were all included in those annotated to the Swiss-Prot and/or Pfam databases. Therefore, the unigenes annotated as being related to the phenylpropanoid, anthocyanin, and monolignol metabolic pathways were selected according to the Swiss-Prot annotations (Table 2).

In order to detect *WD40*, *R2R3-MYB*, *bHLH*, and *Pri-miR828* related sequences in Lollypop, we BLASTed the amino acid sequence of AtTTG1 (WD40 in *Arabidopsis* [60]), the R2R3 repeat motif sequence [51], two lily bHLH (LhbHLH1 and LhbHLH2) [21], and the nucleotide sequence of *VvPri-miR828a*, against the Lollypop sequence database. We used a typical cut off value of E <0.00001 in most case but a cut off value of E <0.001 for *Pri-miR828*, because of the large variation between non-coding RNA sequences.

### qRT-PCR analysis

The total RNA used for qRT-PCR analysis was extracted from upper tepals and tepal bases that were collected at five floral development stages (St 1–5), using a NucleoSpin RNA II Kit (MACHEREY-NAGEL GmbH & Co. KG, Düren, Germany). Total RNA was converted into first strand cDNA using the ReverTra Ace qPCR RT Master Mix with gDNA Remover (Toyobo, Tokyo, Japan). SYBR Premix Ex Taq (TaKaRa) was used to intercalate SYBR Green I into the amplified products, and signals were monitored using the CFX Connect Real-Time system (Bio-Rad, Tokyo, Japan) with the following reaction conditions: preheating at 95 °C for 30 s; 40 cycles of 95 °C for 10 s, 60 °C for 30 s, and 72 °C for 20 s; and a final extension step at 72 °C for 5 min. Primer specificity was confirmed by melting curve analysis, and the amount of *ACTIN* mRNA in each sample was used to normalize the level of each target mRNA. Relative fold-change values of three biological replicates (three different flowers) were used to calculate mean values and standard errors (SE). A post hoc analysis using Tukey’s HSD was performed using R version 3.2.1 (http://cran.r-project.org/doc/manuals/r-release/R-intro.pdf). All primers used for analysis are listed in Additional file 1: Table S3.

## Abbreviations

3GT, anthocyanidin 3-*O*-glucosyltransferase; 3RT, anthocyanidin-3-glucoside rhamnosyltransferase; 4CL, 4-coumaroyl: CoA-ligase; 7GT, anthocyanidin-3-rutinoside 7-glucosyltransferase; ANS, anthocyanidin synthase; C3H, *p*-coumarate 3-hydroxylase; C4H, cinnamate 4-hydroxylase; CAD, cinnamic acid derivative; CHI, chalcone isomerase; CHS, chalcone synthase; DEGs, differentially expressed genes; DFR, dihydroflavonol 4-reductase; F3′H, flavonoid 3′-hydroxylase; F3H, flavanone 3-hydroxylase; FDR, false discovery rates; FLS, flavonol synthase; FPKM, fragments per kb per million mapped reads; GO, gene ontology; GST, glutathione *S*-transferase; HCT, shikimate *O*-hydroxycinnamoyl transferase; HPLC, high-performance liquid chromatography; MATE, multidrug and toxic compound extrusion transporter; MBW complex, R2R3-MYB–bHLH–WDR protein complex; NGS, next-generation sequencing; PAL, phenylalanine ammonia-lyase; pri-miR, primary-microRNA; PTGS, post-transcriptional gene silencing; qRT-PCR, quantitative reverse transcription-PCR; RT, retention time; SE, standard error; SSR, simple sequence repeat; St, floral development stage; TLC, thin layer chromatography

## Additional files

Additional file 1: Table S1. Summary of assembled sequences from the tepal parts of the Asiatic hybrid lily Lollypop. **Table S3.** Primers used for quantitative RT-PCR (qRT-PCR) analysis. **Figure S1.** Phenylpropanoid, anthocyanin, and cinnamic acid derivative biosynthesis pathways in lily tepals. Enzymes whose genes are up-regulated in upper tepals (estimated by qRT-PCR) are shown in blue. 3GT, anthocyanidin 3-*O*-glucosyltransferase; 3RT, anthocyanidin-3-glucoside rhamnosyltransferase; 4CL, 4-coumaroyl: CoA-ligase; 7GT, anthocyanidin-3-rutinoside 7-glucosyltransferase; ANS, anthocyanidin synthase; CHI, chalcone isomerase; CHS, chalcone synthase; C3H, *p*-coumarate 3-hydroxylase; C4H, cinnamate 4-hydroxylase; DFR, dihydroflavonol 4-reductase; F3H, flavanone 3-hydroxylase; F3′H, flavonoid 3′-hydroxylase; FLS, flavonol synthase; GST, glutathione *S*-transferase; HCT, shikimate *O*-hydroxycinnamoyl transferase; MATE, multidrug and toxic compound extrusion transporter; PAL, phenylalanine ammonia-lyase. **Figure S2.** HPLC analysis of anthocyanins and CADs in upper tepals (upper) and tepal bases (basal) of lily cultivars. A: Absorbance at 525 nm (anthocyanins) of the tepal extracts in Lollypop, and cyanidin 3-*O*-glycoside (Cy3G) and cyanidin 3-*O*-rutinoside (Cy3R) standards. B: Absorbance at 340 nm (CADs) of the tepal extracts in six cultivars. **Figure S3.** Alignment of predicted amino acid sequences of isoforms annotated as LhPAL1, LhPAL2, and LhPAL3 (A), and LhCHSa and LhCHSb (B). Letters on black and grey backgrounds indicate identical and similar amino acids, respectively. Asterisks indicate stop codons. **Figure S4.** Relative expression levels of c30288_g1 (*HCT*), c10735_g1 (*MYB3*), c25442_g1 (*MYB8*), c25442_g2, c24227_g1 (*R3-MYB*), c24227_g2 (*R3-MYB*), c18278_g2 (*R3-MYB*), c36339_g1 (*SPL9*), and c16635-g1 (*RCP1*) in upper tepals and tepal bases of Lollypop during floral development (St 1–5). *ACTIN* was used to normalize the expression of target genes. Values and vertical bars indicate the mean ± standard error (n = 3). The same letters above the columns indicate that the values are not statistically significant (*p* <0.05) by Tukey’s HSD. **Figure S5.** Relative expression levels of *LhMYB12* in Sugar Love and *WD40* in Sugar Love and Montreux in upper tepals and tepal bases during floral development (St 1–5, A) and flowers of the cultivars Montreux and Sugar Love (B). *ACTIN* was used to normalize the expression of target genes. Values and vertical bars indicate the means ± standard error (n = 3). The same letters above the columns indicate that the values are not statistically significant (*p* <0.05) by Tukey’s HSD. **Figure S6.** Putative miR828 and pri-miR828 sequences in Lollypop. A: Putative miR828 and its target site appeared in c22900_g1 (*MYB12*). B: Sequence alignment of c13793_g1 and pri-miR828 in *Glycine max* [GmPri-miR828a (NR_126648) and GmPri-miR828b (NR_126651)], *Vitis vinifera* [VvPri-miR828a (NR_127861) and VvPri-miR828b (LM611741)], and *Malus domestica* [MdPri-miR828b (NR_120979) and MdPri-miR828a (NR_120978)]. (PDF 1261 kb) Additional file 2: Table S2. Annotation and expression summary of differentially expressed genes. (XLSX 216 kb)
