# Supplementary material for: RNA-seq-based evaluation of bicolor tepal pigmentation in Asiatic hybrid lilies (Lilium spp.)
Source: BMC Genomics. 2016 Aug 11;17:611. doi: 10.1186/s12864-016-2995-5 (PMC4982199; doi:10.1186/s12864-016-2995-5)
Supplement: Additional file 1: Table S1. — Summary of assembled sequences from the tepal parts of the Asiatic hybrid lily Lollypop. Table S3. Primers used for quantitative RT-PCR (qRT-PCR) analysis. Figure S1. Phenylpropanoid, anthocyanin, and cinnamic acid derivative biosynthesis pathways in lily tepals. Enzymes whose genes are up-regulated in upper tepals (estimated by qRT-PCR) are shown in blue. 3GT, anthocyanidin 3-O-glucosyltransferase; 3RT, anthocyanidin-3-glucoside rhamnosyltransferase; 4CL, 4-coumaroyl: CoA-ligase; 7GT, anthocyanidin-3-rutinoside 7-glucosyltransferase; ANS, anthocyanidin synthase; CHI, chalcone isomerase; CHS, chalcone synthase; C3H, p-coumarate 3-hydroxylase; C4H, cinnamate 4-hydroxylase; DFR, dihydroflavonol 4-reductase; F3H, flavanone 3-hydroxylase; F3′H, flavonoid 3′-hydroxylase; FLS, flavonol synthase; GST, glutathione S-transferase; HCT, shikimate O-hydroxycinnamoyl transferase; MATE, multidrug and toxic compound extrusion transporter; PAL, phenylalanine ammonia-lyase. Figure S2. HPLC analysis of anthocyanins and CADs in upper tepals (upper) and tepal bases (basal) of lily cultivars. A: Absorbance at 525 nm (anthocyanins) of the tepal extracts in Lollypop, and cyanidin 3-O-glycoside (Cy3G) and cyanidin 3-O-rutinoside (Cy3R) standards. B: Absorbance at 340 nm (CADs) of the tepal extracts in six cultivars. Figure S3. Alignment of predicted amino acid sequences of isoforms annotated as LhPAL1, LhPAL2, and LhPAL3 (A), and LhCHSa and LhCHSb (B). Letters on black and grey backgrounds indicate identical and similar amino acids, respectively. Asterisks indicate stop codons. Figure S4. Relative expression levels of c30288_g1 (HCT), c10735_g1 (MYB3), c25442_g1 (MYB8), c25442_g2, c24227_g1 (R3-MYB), c24227_g2 (R3-MYB), c18278_g2 (R3-MYB), c36339_g1 (SPL9), and c16635-g1 (RCP1) in upper tepals and tepal bases of Lollypop during floral development (St 1–5). ACTIN was used to normalize the expression of target genes. Values and vertical bars indicate the mean ± standard error (n = 3). The sa [file 12864_2016_2995_MOESM1_ESM.pdf]

Table S1 Summary of assembled sequences from the tepal parts of the Asiatic hybrid lily Lollypop

	Run score	
	Upper tepals	Tepal bases
Total reads	25,287,406	24,601,874
Total fragments	12,243,532	11,911,846
Clean reads	24,793,322	24,104,276
%	98%	98%

	Assembly score	
	Contigs	Unigenes
Number	49,239	39,426
Total length (bp)	39,694,236	29,260,585
Minimum length (bp)	201	201
Maximum length (bp)	10,772	10,772
Average length (bp)	806	742
Median length (bp)	496	427
N50 length (bp)	1,302	1,228
GC content (%)	47	47

Table S3 Primers used for quantitative RT-PCR (qRT-PCR) analysis

Unigene	Gene	Forward primer (5'-3')	Reverse primer (5'-3')
for MYB, bHLH, and WD40 genes			
c22900_g1	*MYB12*	CGCAAATACGGGCACCACTTCAC	CAGCTTCACCCTGTTTCTTCATCCTCAACC
c2514_g1	*WD40*	CCACTGAATCCTCCGTCGAA	CACGCTCGATGTCCCAGATG
c27198_g1	*bHLH2*	TGAGCAAAGCGAGTTGATGC	GATTGGGGAAATCCGTCACA
c10735_g1	*MYB3*	AAAGCCGCAGGCTTGCTCCGTT	GCGATGAGTGACCATTTGTTACC
c25442_g1	*MYB8*	AGCTTCACAGCCTCCTCGGT	ACTACAGCTGAAACACGGCG
c25442_g2	*MYB8 like*	CGGACAATGAGATCAAGAAC	TATCGCTGCTGCTACTGTGC
c24227_g1	*R3-MYB*	CATCAGAAGAACTTCAAGGAG	AAGTTGTGGCCGATCATTCGG
c24227_g2	*R3-MYB*	CAGAAGAACTTCGAGGAAGAC	GAGGCTGATCATTCGATGGT
c18278_g2	*R3-MYB*	GCTTGGGTCACTCTCCTGAT	CCAGCAATAAGAGCCCATCT
c36339_g1	*SPL9, SPL15*	GACCAGGGTAAACGCAGTTG	CCGTCACGGTATGATGATGA
c16635_g1	*RCP1*	TATTGGCAGCGAAGAGTTCC	CACCGCATCTACAAGGGAAT
for anthocyanin and cinammic acid derivative biosynthesis genes			
c30110_g1_i4	*CHSa*	GCGAAGCTGGGACTGCAGAAGG	CAAGACCACCGTTTCCACGGTT
c30110_g1_i10	*CHSb*	CTGAAGCTGGCGCTGGACAAAAAG	GGTAGTGATCGGAATGCTGTGAAGA
c28136_g1	*CHIa*	CAGTATGGTGTGCAGCTGGA	TGCTCCCACCCAAATACCAC
c28538_g1	*CHIb*	GGGCAACAATACTCCGAGAAGG	CCTTGAGAAGCTCGGAAATG
c28413_g1	*F3H*	GGTGCCTTTGTCGTCAATCT	AACTTCGGTGGGCTTCTTCG
c27194_g1	*F3'H*	ACGCACGACACAAACTTCAG	CGAGTGCTTTCTGGGAAAAG
c30307_g2	*DFR*	AATGGTTGCACCGGTGTGTT	GCACGTTCACAGTTCCAGCA
c26135_g1	*ANS*	GGTGGTGACCAAGATGCTGT	CCAATGTGGACGAGAAGGGA
c35034_g1	*3GT*	GGTTTTTGGACAGGACGAGA	CTCCCGTCACACTCTCCATT
c25725_g1	*3RT*	GACATGAGGAAGGAGGTGGA	CCAACTCGTCTTCCACGATT
c24611_g1	*GST*	GCAAAAGGAGCCCAAATACA	GGTCAGGACCTTGGTCAGAA
c27999_g1	*MATE*	ACCCGTGATTTCTGGAGTTG	ACCCAAATGCAACGAATAGC
c30288_g1	*HCT*	ATTGCGCCTATGGATGACTC	CCTCATAAGCAATCCCTCCA
for normalization of tested genes			
	ACTIN	ATGTATGTTGCAATCCAGGCTGTGC	ATACCAGTAGCTTCCATTCCAACCA

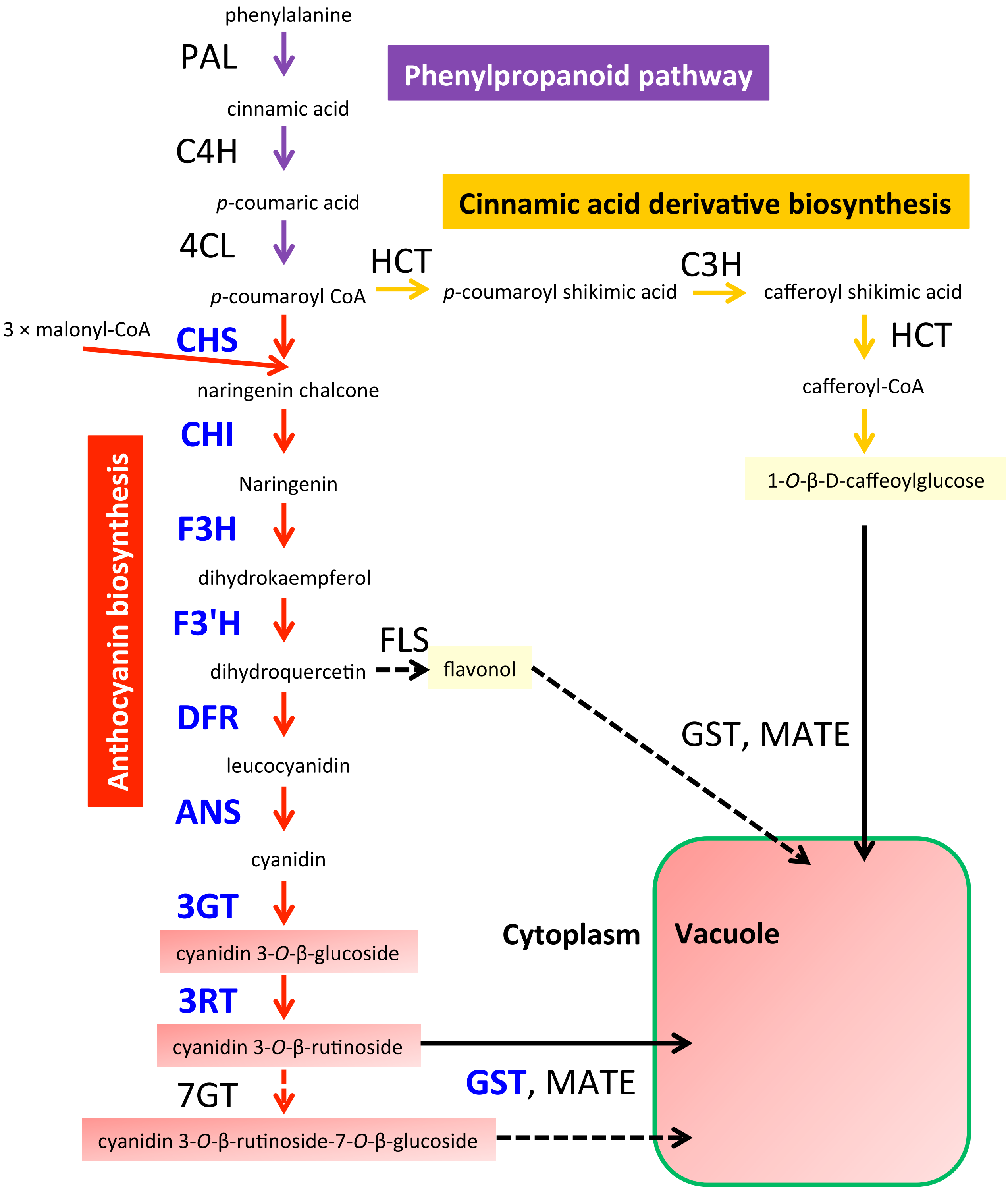

Figure S1. Phenylpropanoid, anthocyanin, and cinnamic acid derivative biosynthesis pathways in lily tepals. Enzymes whose genes are up-regulated in upper tepals (estimated by qRT-PCR) are shown in blue. 3GT, anthocyanidin 3-*O*-glucosyltransferase; 3RT, anthocyanidin-3-glucoside rhamnosyltransferase; 4CL, 4-coumaroyl: CoA-ligase; 7GT, anthocyanidin-3-rutinoside 7-glucosyltransferase; ANS, anthocyanidin synthase; CHI, chalcone isomerase; CHS, chalcone synthase; C3H, *p*-coumarate 3-hydroxylase; C4H, cinnamate 4-hydroxylase; DFR, dihydroflavonol 4-reductase; F3H, flavanone 3-hydroxylase; F3′H, flavonoid 3′-hydroxylase; FLS, flavonol synthase; GST, glutathione *S*-transferase; HCT, shikimate *O*-hydroxycinnamoyl transferase; MATE, multidrug and toxic compound extrusion transporter; PAL, phenylalanine ammonia-lyase.

A

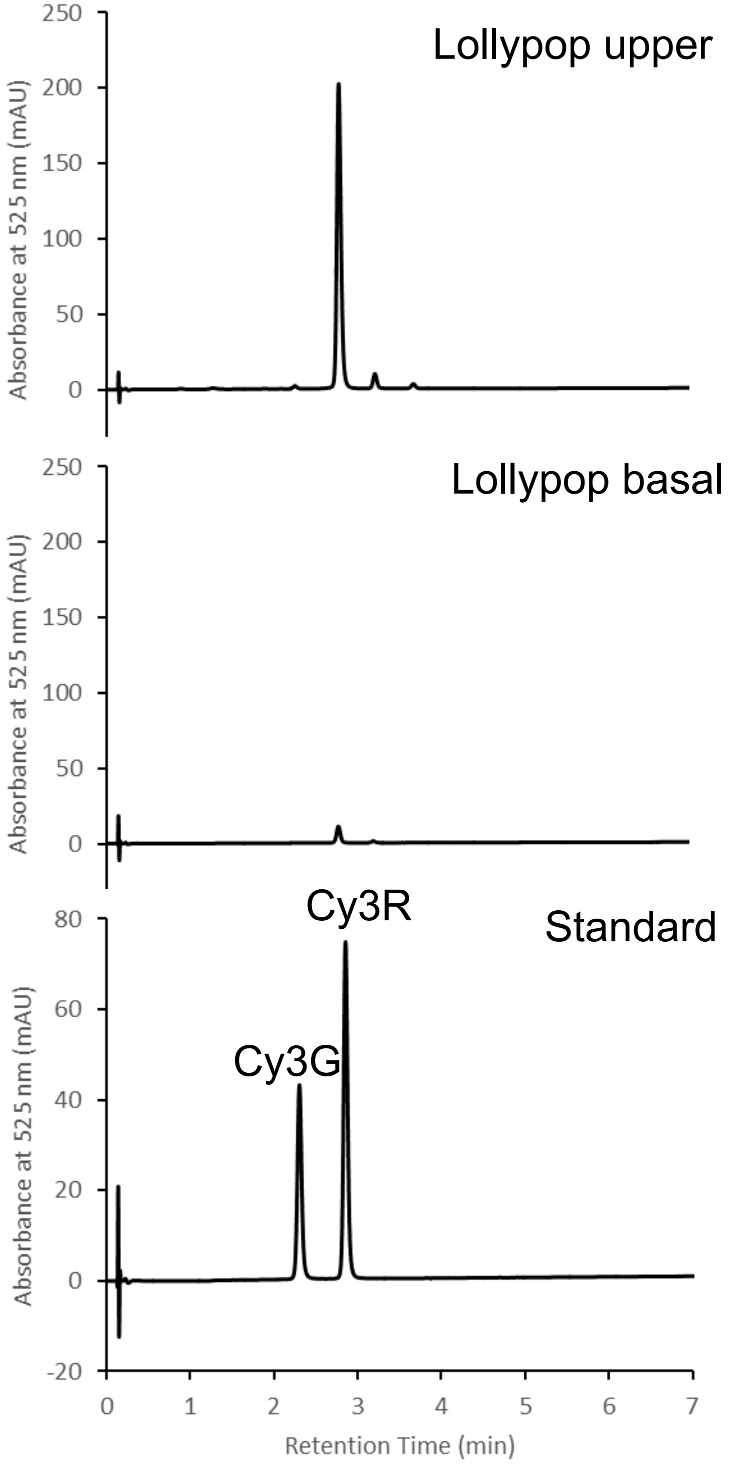

Lollypop upper
Absorbance at 525 nm (mAU)
250
200
150
100
50
0
Lollypop basal
Absorbance at 525 nm (mAU)
250
200
150
100
50
0
Cy3R
Standard
Cy3G
Absorbance at 525 nm (mAU)
80
60
40
20
0
-20
0
1
2
3
4
5
6
7
Retention Time (min)

(Fig. S2)

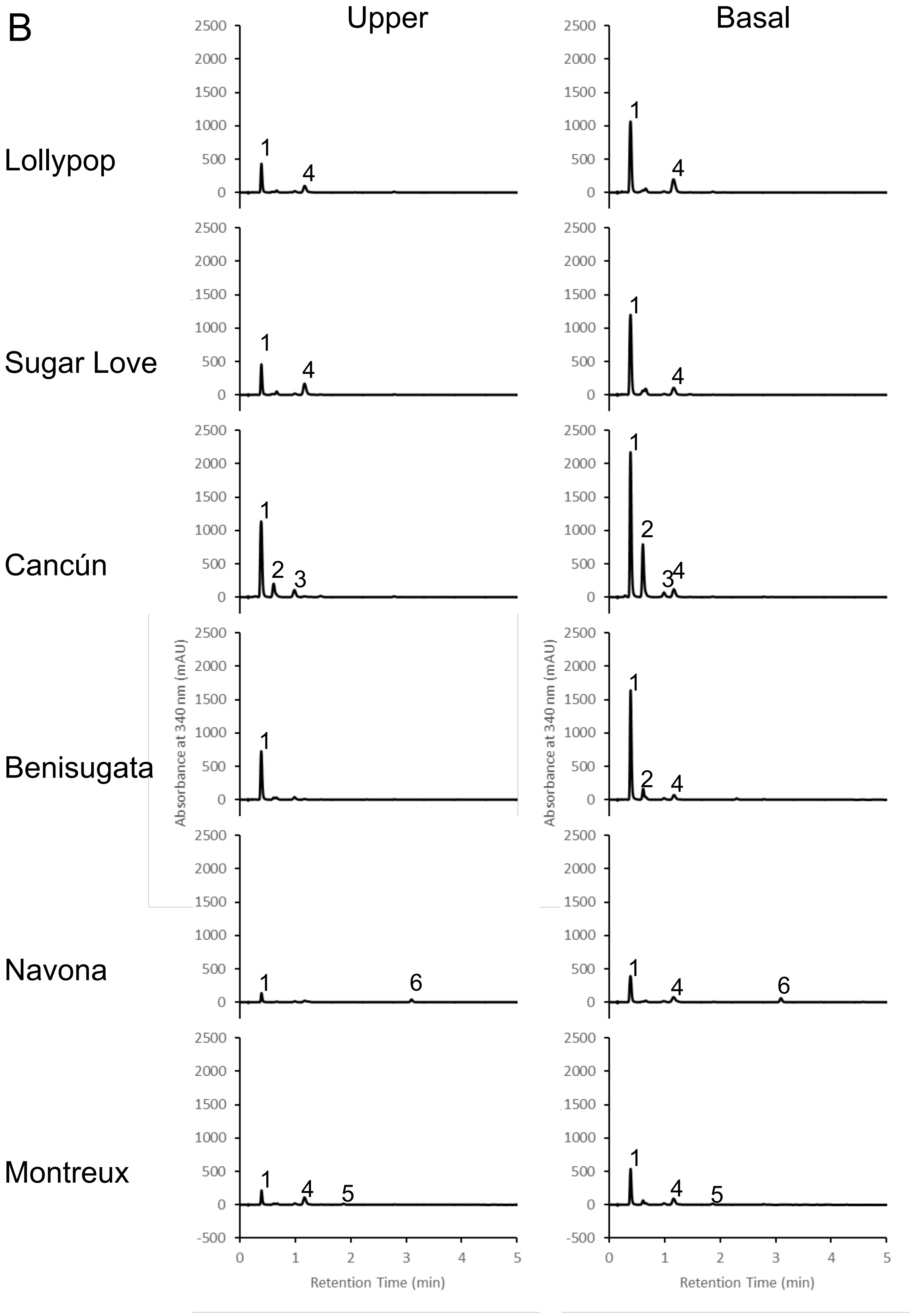

Figure S2. HPLC analysis of anthocyanins and CADs in upper tepals (upper) and tepal bases (basal) of lily cultivars. A: Absorbance at 525 nm (anthocyanins) of the tepal extracts in Lollypop, and cyanidin 3-*O*-glycoside (Cy3G) and cyanidin 3-*O*-rutinoside (Cy3R) standards. B: Absorbance at 340 nm (CADs) of the tepal extracts in six cultivars.

(A) PAL

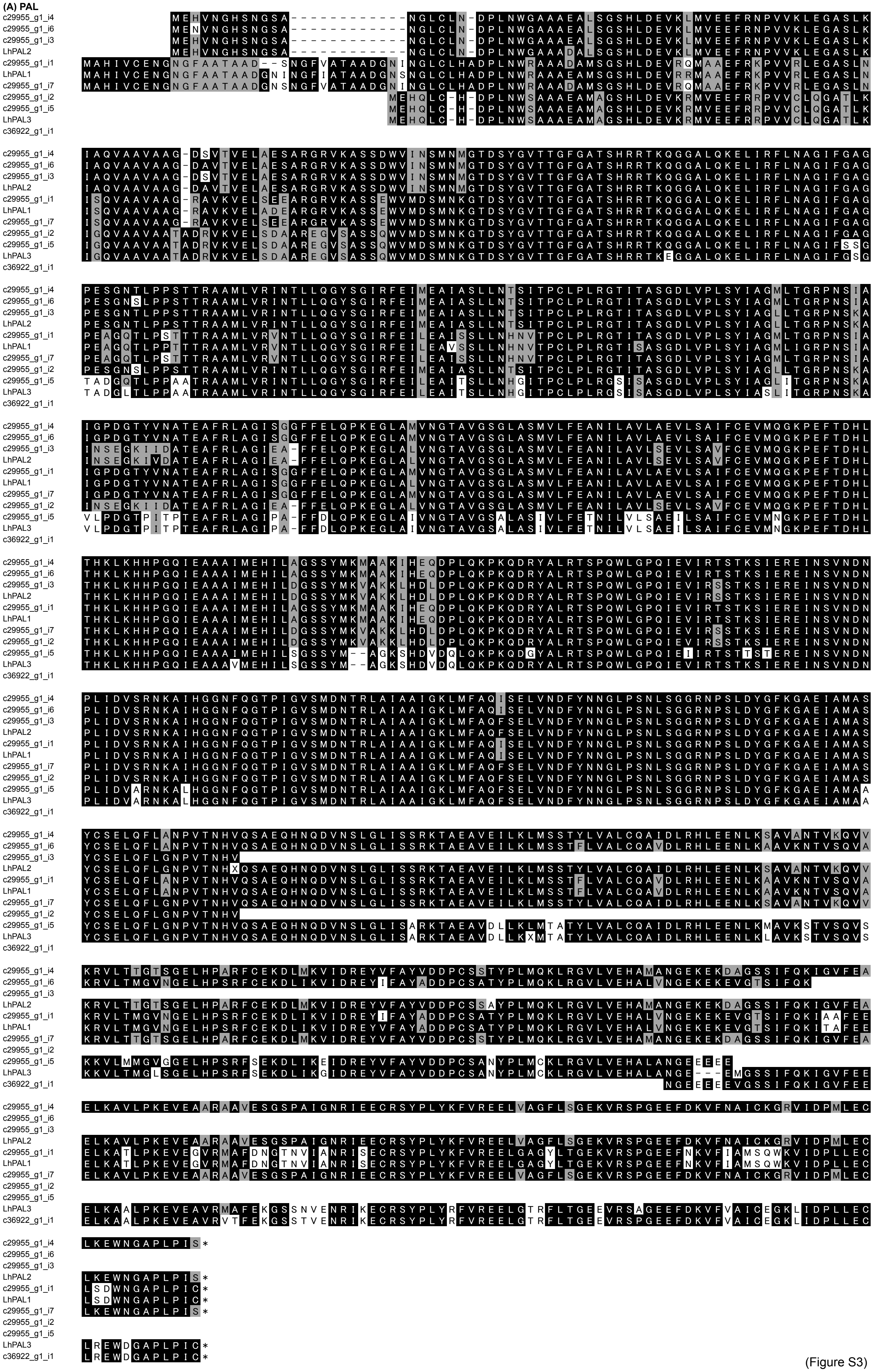

(Figure S3)

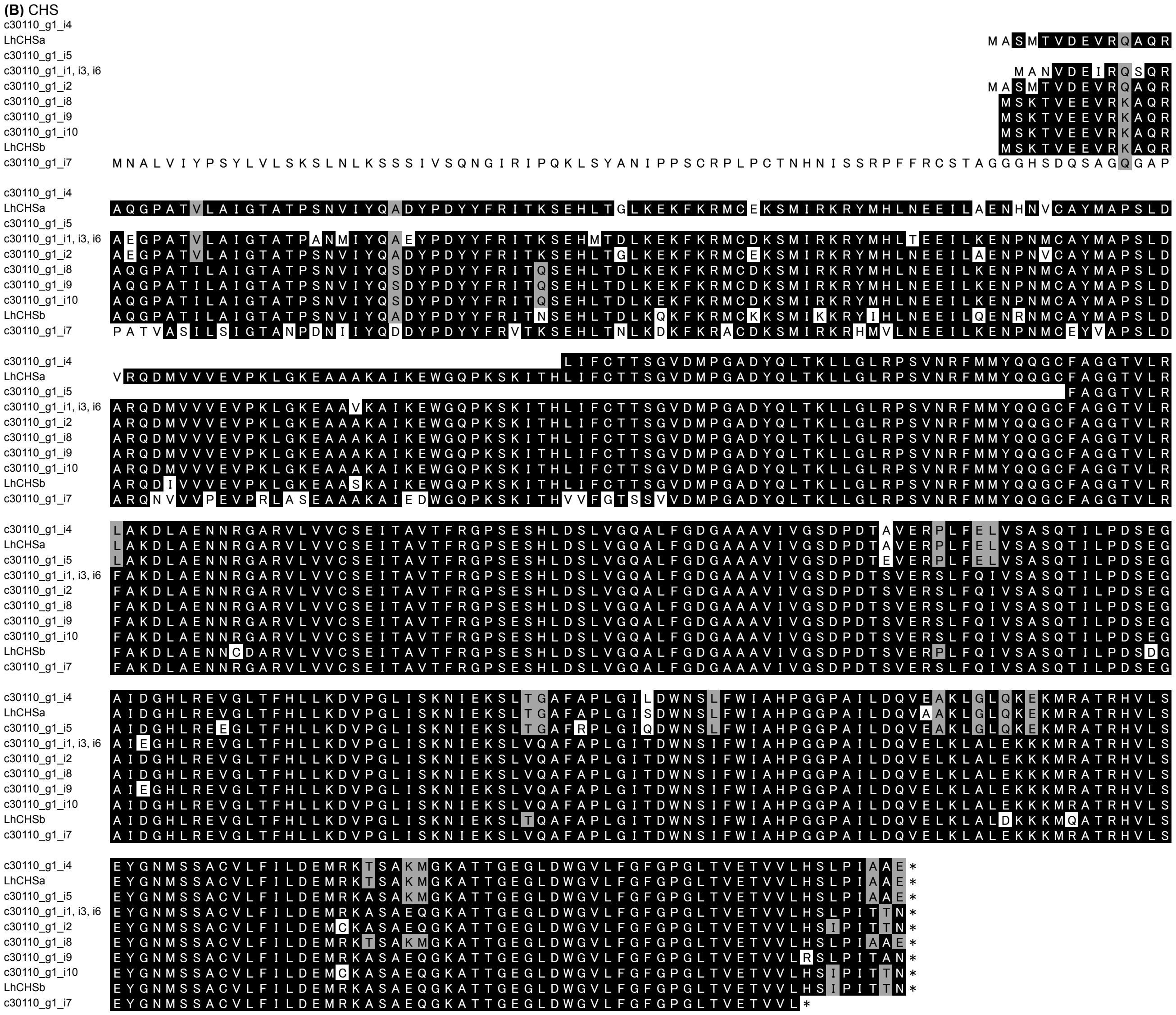

Figure S3. Alignment of predicted amino acid sequences of isoforms annotated as LhPAL1, LhPAL2, and LhPAL3 (A), and LhCHSa and LhCHSb (B). Letters on black and grey backgrounds indicate identical and similar amino acids, respectively. Asterisks indicate stop codons.

(A) Three PALs (LhPAL1, LhPAL2, and LhPAL3) have been identified in Montreux [10]. Of the seven isoforms of c29955_g1, two, three, and two exhibited high similarities to LhPAL1, LhPAL2, and LhPAL3, respectively. Since the sequences of the three PALs are similar to each other [10], the seven isoforms of c29955_g1 should be classified into a single unigene (i.e., the three *PAL* gene sequences were not distinguished during *de novo* assembly). A unigene c36922_g1, which only consisted of 114 amino acid residues, also exhibited a high similarity to the LhPAL3 of Montreux (Table 1).

(B) Each of the 10 isoforms of c30110_g1 was similar to either LhCHSa or LhCHSb from Montreux. Among three CHSs that have been isolated in Montreux tepals, the sequences of LhCHSa and LhCHSb are highly similar [31].

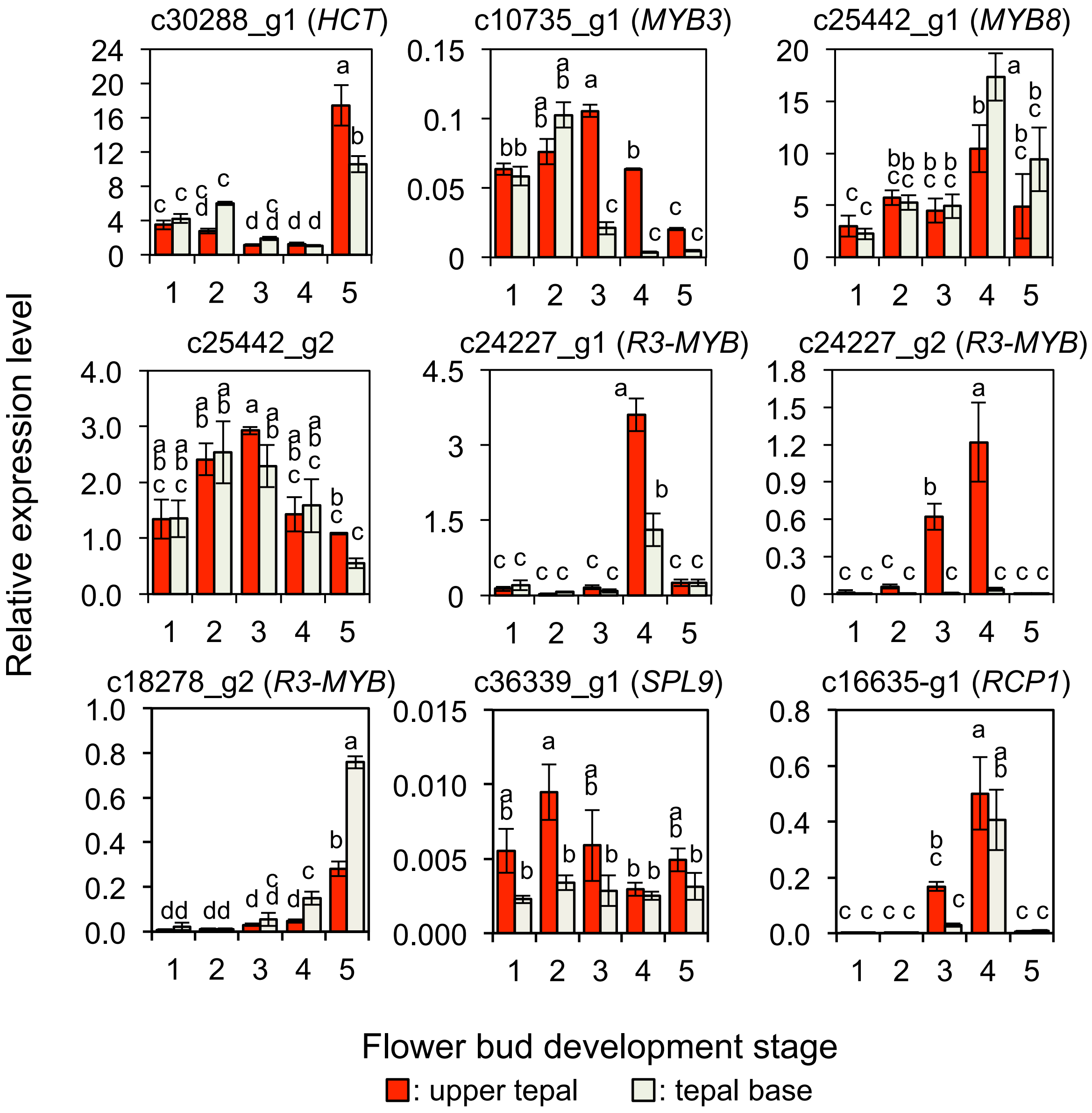

Figure S4. Relative expression levels of c30288_g1 (*HCT*), c10735_g1 (*MYB3*), c25442_g1 (*MYB8*), c25442_g2, c24227_g1 (*R3-MYB*), c24227_g2 (*R3-MYB*), c18278_g2 (*R3-MYB*), c36339_g1 (*SPL9*), and c16635-g1 (*RCP1*) in upper tepals and tepal bases of Lollypop during floral development (St 1–5). *ACTIN* was used to normalize the expression of target genes. Values and vertical bars indicate the mean ± standard error (n = 3). The same letters above the columns indicate that the values are not statistically significant ($p < 0.05$) by Tukey's HSD.

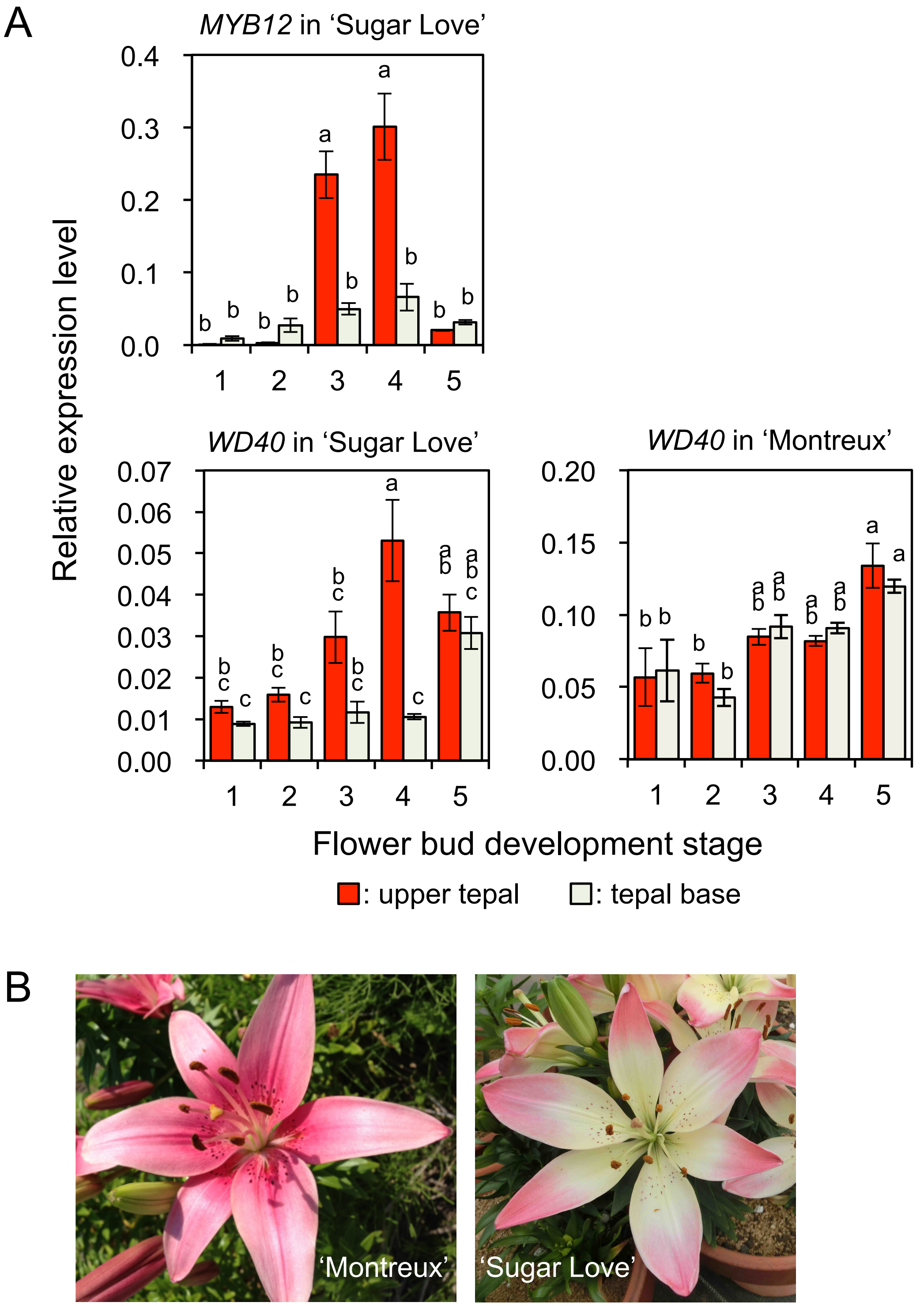

Figure S5. Relative expression levels of *LhMYB12* in Sugar Love and *WD40* in Sugar Love and Montreux in upper tepals and tepal bases during floral development (St 1–5, A) and flowers of the cultivars Montreux and Sugar Love (B). *ACTIN* was used to normalize the expression of target genes. Values and vertical bars indicate the means ± standard error (n = 3). The same letters above the columns indicate that the values are not statistically significant (p <0.05) by Tukey's HSD.

A

putative miR828 target site in c22900_g1 (*MYB12*)

```
5'-...CCCGGUCGGACAGCUAAUGAUAUUAAGAACUACUGGAAUUCUCACUUGAGCAAGAGAAAAGUGAAUGUCGA...-3'
                                    3'-ACCUUAUGAGUAAACUCGUUCU-5'
```

putative miR828 (22 nt)

B

putative miR828

```
MdPri-miR828b   -------UCCUCUUCGUAAUGUUUCUUGCUCAAAUGAGUAUUCCAGCCACAGUUUGCAUGCAGCUUGCAUAUAUAUAUAUAAG
MdPri-miR828a   -------UCCUCUUUGUAAUGUUUCUUGCUCAAAUGAGUAUUCCAGCCACAGUUUGCCUGCAGCUUGCAUAUAUAUAUAUA--
GmPri-miR828b   -------------UUGUAAUGUUUCUUGCUCAAAUGAGUAUUCCAACCACAGUC-----ACAUUUGGCAUGAAU---------
GmPri-miR828a   -------------UUGUAAUGUUUCUUGCUCAAAUGAGUAUUCCAACCACAGUC-----ACAUUUGGCAUGAAUAU-------
VvPri-miR828b   --------CCUCUUUGUAAUGUUUCUUGCUCAAAUGAGUAUUCCAACAACAGUA-----GCCUUUUGAACAAGAACAUAUGUU
VvPri-miR828A   --------------UAUAAAGUUUCUUGCUCAAAUGAGUAUUCCAA--ACAACA-----GCUUGUGAAGCAAUGAUU------
c13793_g1       UUUUCCUCCCACAUGGGAAAGCCUCUUGCUCAAAUGAGUAUUCCAUCUAUGGUGCAGAAGCUUUGGGUCCU------------
                                 ** *  **********************   *           *

MdPri-miR828b   AACAUGUAUGUGUAUAUAUAUAUAGCACUGGUGCUUCUGCAAUCCUGAGAUGCUCAUUUGGGCAAGCAACGUUACAAGAGGA
MdPri-miR828a   --------------UAUAUAUAUAGCGCUGGUGCUUCUGCAAUUCUGAGAUGCUCAUUUGAGCAAGCAGCGUUACAAGAGGA
GmPri-miR828b   -------------------GUGUGCCACUG----UCCCCAAAGGCUGAGAUGCUCAUUUGAGCAAGCAAUGUUAAAA
GmPri-miR828a   ------------------UGUGUGCCACUG----UCCUCAAAGGAUGAGAUGCUCAUUUGACCAAGCAAUGUUAAAA
VvPri-miR828b   UUUGCCU--UGUAUGGUUAUGAUGUUGCUGGUA-UUUACUGUUUCUGAGAUGCUCAUUCGAGCAAGAAAUAUUAGAAGAGG
VvPri-miR828A   --------------ACUGUCAUAGUCUCUGCAUGUGGAUUGUUGUUGAGAUGCUCAUUUGAGGAAGCAACCUUAAAAAUG
c13793_g1       --------------------CUCGGAGCUUCAUGCAUUGCAGAAGUG-GAUGCUCAUUAGGGUAAGAUGCAUUCUGUGGGA
                                           **                 ** ********** *   ***     **

c13793_g1       GGAAGAAGAGAUAUUCAAAUGGAUGGCCAACAGGUUCUCAGCAGCAUAACUCUCUGAUUUCUCCGCUUAUUUUGGUAGCC
                UAUGCUUGCCUGAGUGCCCCUCUGGUACGAGUGAGCCCCCAUCCCCUCCAUGCAAAAUCCCUCCCCGACUUAUCAUAUACC
                UUCAUCUUGUUAAUUGUUUCUGUGUCUCCUCCUCCAGCUCCAUAUCGAGACAAUCACACAGCCCACACCUGCGCCGACAC
                UGAGGCAAGCAGUGCCGCCCUCCAGCUACUCAGCAUCUCCUU
```

Figure S6. Putative miR828 and pri-miR828 sequences in Lollypop. A: Putative miR828 and its target site appeared in c22900_g1 (*MYB12*). B: Sequence alignment of c13793_g1 and pri-miR828 in *Glycine max* [GmPri-miR828a (NR_126648) and GmPri-miR828b (NR_126651)], *Vitis vinifera* [VvPri-miR828a (NR_127861) and VvPri-miR828b (LM611741)], and *Malus domestica* [MdPri-miR828b (NR_120979) and MdPri-miR828a (NR_120978)].
